# Effects of green exercise on mental health: a systematic review and meta-analysis

**DOI:** 10.3389/fpsyg.2026.1802759

**Published:** 2026-04-14

**Authors:** Xinyi Liu, Zongyi Sun, Xiaolin Wang, Delong Dong, Shamsulariffin Bin Samsudin

**Affiliations:** 1 Department of Physical Education, Ludong University, Yantai, China; 2 Yantai Economic and Technological Development Zone Fourth Junior Middle School, Yantai, China; 3 Faculty of Educational Studies, University Putra Malaysia, Serdang, Malaysia

**Keywords:** green exercise, green space, mental health, meta-analysis, wellbeing

## Abstract

**Introduction:**

With the increasing global prevalence of mental health issues, green exercise has emerged as a promising intervention, though its effectiveness remains uncertain. This systematic review and meta-analysis aims to clarify the mental health benefits of green exercise by comparing its impact on well-being, positive affect, and negative affect with indoor exercise, built-up exercise, and non-exercise groups.

**Methods:**

Fifty-one relevant studies, the outcome data of which were used for the systematic review and meta-analysis, were identified across the PubMed, Web of Science, EBSCOhost, PsycINFO, and the Cochrane Central Register of Controlled Trials databases.

**Results:**

The meta-analysis revealed significant effects of green exercise on mental health outcomes. Compared to non-exercise, green exercise significantly improved well-being (SMD = 0.46), positive affect (SMD = [1.18, 1.83]), and reduced negative affect (SMD = [-0.34, -3.38]). Compared to indoor exercise, it significantly improved well-being (SMD = 0.65), positive affect (SMD = [0.68, 1.20]), and slightly reduced negative affect (SMD = [-0.31, -1.38]). Compared to built-up exercise, it significantly improved well-being (SMD = 0.22), had a large effect on positive affect (SMD = [0.59, 1.01]), and slightly reduced negative affect (SMD = [-0.21, -0.57]).

**Conclusion:**

Green exercise significantly improves mental well-being, with superior effects on positive affect and reduction of negative affect compared to indoor exercise, built-up exercise, and non-exercise. Future research should explore the long-term effects of green exercise and identify optimal conditions for maximizing its mental health benefits.

**Syestematic review registration:**

https://www.crd.york.ac.uk/PROSPERO/view/CRD420251235047, Registration ID (Unique Identifier): CRD420251235047.

## Introduction

Mental health issues have become a significant global challenge, affecting nearly 1 billion people worldwide, with depression and anxiety being the most prevalent conditions, according to the World Health Organization (2022). These conditions contribute to a diminished quality of life, impaired social and occupational functioning, and an increased risk of comorbid chronic diseases (Gabarrell-Pascuet et al., 2023; Hohls et al., 2021; Li Z. et al., 2022). The growing burden of mental illness underscores the urgent need for effective interventions, particularly in the context of modern urbanization, which has been associated with a rise in mental health problems (Zhang D. et al., 2023; Zhang Z. et al., 2023). Current strategies, including pharmacological treatments, psychotherapy, and community-based interventions, have proven effective (Heinz and Liu, 2022; Wang et al., 2023; Zhang et al., 2025). However, they are often hindered by challenges such as accessibility, adherence, and potential side effects (Bian et al., 2023; Marrero et al., 2020; Oliva et al., 2021). These barriers complicate large-scale, long-term implementation, underscoring the need for low-risk, sustainable mental health interventions that can reach diverse populations.

Green exercise has gained significant attention due to its potential to enhance both physical and mental health, offering an affordable and accessible intervention (Coventry et al., 2021; Firnhaber et al., 2025; Hu et al., 2025). It encompasses any physical activity performed in outdoor environments with substantial natural elements, such as parks or forests (Brito et al., 2022). However, not all outdoor activities are classified as green exercise. For instance, exercise on urban streets with limited or no greenery is considered built-up exercise in this study, which refers to physical activity performed in highly urbanized areas with minimal natural green spaces. Green exercise embodies the intersection of physical activity and the natural environment, with several theoretical frameworks supporting its mental health benefits. The biophilia hypothesis proposes that humans possess an innate, evolutionarily grounded tendency to affiliate with nature, suggesting that natural environments are inherently restorative (Wilson, 2017). Attention Restoration Theory articulates a cognitive mechanism: natural environments restore directed attention, a resource depleted by prolonged focus, by engaging involuntary attention through softly fascinating stimuli such as rustling leaves or flowing water (Joye and Dewitte, 2018). Stress Reduction Theory complements this by emphasizing affective and physiological pathways, arguing that natural settings evoke positive emotions and rapidly reduce physiological stress markers like heart rate and cortisol (Ulrich et al., 1991). Together, these theoretical perspectives provide a foundation for expecting green exercise to benefit mental health, though they were developed primarily to explain passive nature exposure rather than active physical activity in natural settings.

While the potential mental health benefits of green exercise are promising, empirical findings on the mental health benefits of green exercise remain inconsistent (Dickmeyer et al., 2025; Hvid et al., 2025; Park et al., 2024; Trammell and Aguilar, 2021). Previous studies have synthesized this literature, reporting mixed findings regarding the effects of green exercise on mental health, which may be due to methodological and conceptual limitations (Brito et al., 2022; Firnhaber et al., 2025; Hu et al., 2025; Li H. et al., 2022). A common limitation is the emphasis on general mental health indicators rather than specific outcomes such as anxiety, depression, or stress, which may obscure outcome-specific effects (Firnhaber et al., 2025; Hu et al., 2025). Earlier syntheses also included limited primary studies, weakening statistical power and the stability of effect estimates (Hu et al., 2025; Li H. et al., 2022). The operationalization of green exercise has been inconsistent, often incorporating built-up or mixed environments, which can dilute the unique contribution of natural settings (Brito et al., 2022; Hu et al., 2025). Furthermore, undistinguished comparators (e.g., non-exercise, and indoor exercise groups) confound attribution of effects to natural environments (Hu et al., 2025). Comparisons with such control conditions, such as non-exercise, indoor exercise, and built-up exercise groups, are necessary to isolate whether mental health benefits derive from physical activity alone, the natural environment alone, or their combination. Different types of physical activity conducted in green spaces may also produce varying mental health effects, an important consideration for understanding the full scope of green exercise benefits. Addressing these limitations is therefore essential for advancing understanding of green exercise’s mental health effects.

This systematic review and meta-analysis aims to provide a more precise and comprehensive evaluation of the mental health effects of green exercise. Unlike previous reviews, it incorporates a larger evidence base, distinguishes green exercise more clearly from built-up exercise, separates non-exercise, indoor exercise, and built-up exercise comparators, and examines specific mental health outcomes rather than broad indicators alone. This approach provides a more nuanced understanding of the mental health effects of green exercise and may inform evidence-based interventions and public health strategies to promote mental wellbeing.

## Methods

### Search strategy and study selection

**Figure 1:**
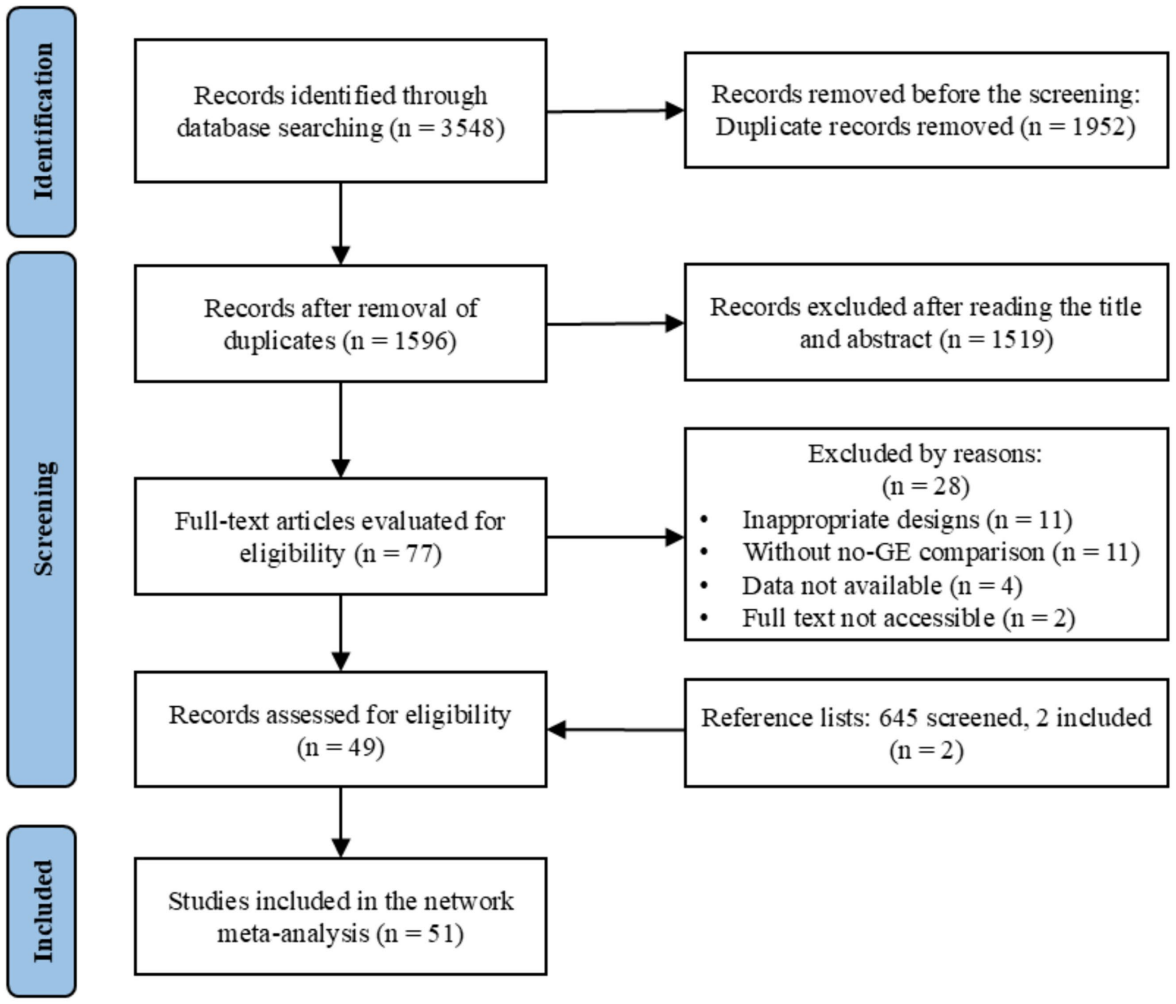
PRISMA flow diagram.

This systematic review and meta-analysis were registered in the PROSPERO, with protocol number CRD420251235047, and conducted in accordance with the updated PRISMA guidelines. A comprehensive electronic search was conducted to identify articles published by November 7, 2025, across PubMed, Web of Science, EBSCOhost, PsycINFO, and the Cochrane Central Register of Controlled Trials. The search employed a variety of relevant terms to capture all potentially eligible studies on green exercise interventions for mental health (for the full list of search terms, see Supplementary File S1). After duplicates were removed, the titles of the remaining articles were reviewed, followed by the examination of their abstracts and full texts. Additionally, reference lists from relevant systematic reviews and meta-analyses were screened to identify further eligible studies. When full texts could not be retrieved, corresponding authors were contacted (up to two attempts). Studies were excluded only if full texts remained unavailable. The study selection process is shown in Figure 1. Screening was performed independently by two researchers, with high inter-reviewer agreement (*κ* = 0.882; overall agreement rate = 94.1%). All discrepancies were resolved through discussion with a third researcher.

### Eligibility criteria

The eligibility criteria were required to meet the following PICOS framework (Population, Intervention, Comparison, Outcome, Study design): (1) Participants aged 18 years or older, with no underlying diseases. (2) The intervention involved green exercise, conducted in natural environments incorporating green plants. (3) The comparison groups included indoor exercise groups, build-up exercise groups (urban outdoor environments with minimal greenery or no greenery), or non-exercise control groups. (4) Primary outcomes were mental health, conceptualized as three distinct dimensions: wellbeing (e.g., quality of life, flourishing), positive affect (e.g., vigor, calm), and negative affect (e.g., anxiety, depression, stress). This three-dimensional framework is consistent with dual-factor models that distinguish between wellbeing and psychopathology (Magalhães, 2024). Studies reporting only physiological or cognitive outcomes without an affective component were excluded. (5) Only randomized controlled trials (RCTs) or crossover RCTs, published in peer-reviewed journals, were included.

### Data extraction

Data extraction was conducted by two independent researchers, with discrepancies resolved by a third researcher. The data extracted from the included studies included mental health outcomes, which were categorized into wellbeing, positive affect (e.g., overall positive affect, calm, vigor), and negative affect (e.g., overall negative affect, stress, depression, anxiety, anger, fatigue, confusion). The primary data extracted included the mean and standard deviation for pre- and post-intervention measures in both the experimental and control groups. Additionally, demographic and study characteristics were extracted, including sample size, participant age, gender, region, exercise interventions, and measured outcome variables. For detailed information on the extracted data, see Table 1.

**Table 1:** Characteristics of the included studies.

Studies	Design	Groups	Female %	Mean age	Exercise interventions	Outcome variables
Anandh et al. (2021)	RCT	GE 52 NE 52	55%	69 ± 2.7	20 min mindful walking in trees and fields vs. routine control	Positive affect, negative affect
Anzman-Frasca et al. (2023)	RCT	GE 26 IE 15	93%	48.3 ± 11.1	Hiking challenge program vs. indoor activity	Stress
Bang et al. (2017)	RCT	GE 51 NE 48	53%	24.3 ± 4.2	40 min forest walking vs. routine control	Stress management, depression
Barton et al. (2012)	RCT	GE 24 IE 14 NE 15	62%	43.4 ± 12.2	Walking in green spaces vs. swimming vs. social activity	Self-esteem, overall mood
Berman et al. (2012)	Crossover-RCT	GE 19 BUE 19	60%	26	50-55 min walking in the park vs. in built-up areas	Positive affect, negative affect
Bodin and Hartig (2003)	Crossover-RCT	GE 12 BUE 12	50%	38.4 ± 6.6	1 h running along a wooded lake shore vs. in manor and estate buildings	Revitalization, tranquility, anxiety/depression, anger
Bramwell et al. (2023)	RCT	GE 7 IE 6	69%	23.6 ± 5.3	Green outdoor exercise vs. indoor exercise	Stress
Brown et al. (2014)	RCT	GE 27 BUE 27 NE 19	21%	42.0 ± 10.6	20 min walking in green spaces vs. in built-up areas vs. waiting control group	Mental health
Byrka and Ryczko (2018)	Crossover-RCT	IE 36 GE 28	80%	29.4 ± 8.9	40 min salsa-solo in a park with trees or in a dance room	Positive emotions
Calogiuri et al. (2018)	Crossover-RCT	IE 26 GE 26 NE 26	46%	26 ± 8	Outdoor walking vs. treadmill walking vs. sedentary activity	Positive affect, negative affect, tranquility, fatigue
Carter et al. (2022)	RCT	GE 18 IE 18 NE 18	61%	20.4 ± 2.4	20 min walking or running around campus green space vs. on a treadmill vs. no exercise	Stress, anxiety
Crossan and Salmoni (2021)	Crossover-RCT	GE 22 IE 22	59%	23	10 min treadmill walking with a simulated green scenery vs. white background	Total positive and negative affect
de Brito et al. (2019)	Crossover-RCT	GE11 BUE12	83%	49.7 ± 6.5	50-min walking in an arboretum vs. in a residential development area	Positive affect, negative affect
Dickmeyer et al. (2025)	RCT	GE 20 NE 17	0%	44.1 ± 15.8	4 km bushwalk vs. indoor therapy	Overall psychological distress, anxiety, mental wellbeing, psychological flexibility
Elsadek et al. (2019)	Crossover-RCT	GE 364 BUE 364	45	23 ± 4.6	15 min walking in green spaces or in urban buildings	Depression, anger and hostility, fatigue, confusions, vigor, total mood disturbance
Fuegen and Breitenbecher (2018)	Crossover- RCT	GE 36 IE 36	60%	21.6 ± 7.7	15 min treadmill walking in green spaces vs. virtual green spaces	Positive affect, negative affect, energy, tiredness
Geniole et al. (2016)	Crossover-RCT	GE 31 BUE 31	0%	24.6 ± 3.9	15 ± 3 min walking in a naturalized landfill vs. urban areas	Positive affect, arousal
Gidlow et al. (2016)	Crossover-RCT	BUE 38 GE38	40%	40.9 ± 17.6	30 min walking in a country park vs. residential streets	Total mood disturbance
Harte and Eifert (1995)	Crossover-RCT	GE 10 IE 10 NE 10	0%	27.1	12 km green running vs. 45 min treadmill running vs. reading magazines	Tension/anxiety, depression/rejection, anger/hostility, confusion, vigor/activity, fatigue
Hartig et al. (2003)	Crossover-RCT	GE 28 BUE 28	50%	20.9	50 min nature walking vs. urban walking	Positive affect, anger
Hvid et al. (2025)	Crossover-RCT	GE 22 NE 23	79%	51.1 ± 10.6	2-40 min forest walking vs. waitlist control group	Wellbeing, fatigue
Jang and So (2017)	Crossover-RCT	GE 9 IE 9	22%	22.6 ± 1.0	40 min reverse turning kicks in a natural environment vs. in building areas	Tension and anxiety, depression, anger and hostility, vigor, fatigue, confusion
Johansson et al. (2011)	Crossover-RCT	GE 10 BUE 10	50%	23.3 ± 2.9	40 min park walking vs. street walking	Revitalization, tranquility, fatigue, anxiety/depression, anger
Keenan et al. (2021)	RCT	GE 25 BUE 25	60%	40.3 ± 12.7	30 min walking in nature environments vs. urban environments	Wellbeing, positive affect, negative affect
Kerr et al. (2006)	Crossover-RCT	GE 22 IE 22	0%	20.7 ± 1.5	5 km running on a tree-lined footpath vs. on a lab treadmill	Overall pleasant, relaxation, anxiety, excitement, boredom, placidity, anger, tension stress
Kinnafick and Thøgersen-Ntoumani (2014)	Crossover-RCT	GE 10 BUE 10 IE 10 NE 10	80%	23.0 ± 7.7	15 min walking in green spaces vs. urban environments vs. indoor environments vs. sitting in a room	Positive affect, negative affect, energy, tiredness, tension, calmness
Klaperski et al. (2019)	Crossover-RCT	GE 90 IE 50	49%	24.4 ± 3.6	Outdoor exercise classes along the river and forest vs. indoor exercise classes	Negative affect, stress, anxiety
Lee et al. (2014)	Crossover-RCT	GE 24 BUE 24	0%	21.1 ± 1.2	12–15 min forest walking vs. urban walking	Tension-anxiety, depression-dejection, anger-hostility, fatigue, confusion, vigor
Legrand et al. (2022)	Crossover-RCT	GE 50 BUE 50 NE 50	31%	20.2 ± 1.3	30 min walking in a tree/bush walkway or in residential areas	Positive affect, negative affect
Levinger et al. (2023)	RCT	GE 8 NE 8	88%	85.4 ± 5.3	1.5 h structured supervised exercise program at park vs. usual care programs	Depression
Li et al. (2021)	RCT	GE 24 BUE 24	54%	54.6 ± 2.6	1.6 km walking in a green space vs. in an urban area	Positive affect, negative affect
Ma et al. (2023)	RCT	GE 50 BUE 49	90%	23.6 ± 2.2	35 min walking in forest areas vs. city areas	Curiosity, total mood disturbance
Mao et al. (2012)	Crossover-RCT	GE 10 BUE 10	0%	20.1 ± 0.5	1.5 h walking in forest areas vs. city areas	Anxiety, depression, anger, vigor, fatigue, confusion
Marselle et al. (2013)	RCT	GE 216 BUE 44	62%	55+	Walking in the country park, nature reserve vs. streets, shopping centers, plaza	Mental wellbeing, depression, stress, positive affect, negative affect
Sales et al. (2017)	RCT	GE 27 NE 21	70%	73.0 ± 8.1	Strength, balance, coordination, mobility and flexibility training in the park vs. daily activities	Mental health
Menzel et al. (2020)	Crossover-RCT	GE 20 BUE 20	0%	23.6 ± 2.8	22 min walking at forest park vs. along city streets	Stress
Niedermeier et al. (2017)	Crossover-RCT	GE 42 IE 42 NE 42	48%	32.0 ± 4.8	6 km mountain hiking vs. 1.5 h indoor treadmill walking vs. sedentary control situation	Activation, elation, calmness, fatigue, depression, anger, excitement, anxiety
Nisbet and Zelenski (2011)	Crossover-RCT	GE 20 IE 24	57%	20.8 ± 5	17-min walking experience in a green corridor versus through tunnels	Positive affect, negative affect
Ojala et al. (2019)	Crossover-RCT	GE 83 BUE 83	100%	47.6 ± 8.6	30 min walking in the forest vs. in built-up environments	Restoration, vigor
Olafsdottir et al. (2020)	RCT	GE 20 IE 24	69%	24.4 ± 2.6	Walking in green spaces or in a gym vs. no exercise	Negative affect, positive affect
Park et al. (2007)	Crossover-RCT	GE 6 BUE 6	0%	22.8 ± 1.4	20 min walking in the forest vs. in city areas	Comfort, calm
Park et al. (2009)	Crossover-RCT	GE 12 BUE 12	0%	21.8 ± 0.8	20 min walking in the forest vs. in city areas	Comfort, calm, refreshment
Park et al. (2024)	RCT	GE 36 NE 27	86%	53.8 ± 9.9	Forest healing program vs. no intervention	Mood disturbance, distress
Shin et al. (2013)	RCT	GE 38 IE 31	100%	20.5 ± 1.5	35 min walking in the forest vs. in the gym	Anxiety, satisfaction, positive emotion, negative emotion, happiness
Takayama et al. (2014)	Crossover-RCT	GE 45 BUE 45	0%	21.1 ± 1.2	15 min walking in forest environments vs. urban environments	Tension and anxiety, depression, anger and hostility, vigor, fatigue, confusion
Teas et al. (2007)	Crossover-RCT	GE 19 IE 19	100%	58 ± 4	1 h walking in a grassy area vs. in a campus gym	Pleasant, anger, anxious
Trammell and Aguilar (2021)	Crossover-RCT	GE 28 IE 28	54%	27.0 ± 10.2	20 min running in a parking area vs. in a lab	Happiness, stress, positive affect, negative affect
Turner and Stevinson (2017)	Crossover-RCT	GE 22 IE 22	36%	33 ± 8.3	6 km running in outdoor green environments vs. in an indoor gym	Activation
Tyrväinen et al. (2014)	Crossover-RCT	GE 77 BUE 77	92%	47.6 ± 8.7	30 min walking in the forest vs. in built-up environments	Restoration, vitality, positive affect, negative affect
Watkins-Martin et al. (2022)	RCT	GE 20 BUE 17	68%	49.3 ± 11.0	60 min nature walking vs. urban walking	Negative affect, positive affect
Zhang D. et al. (2023)	Crossover-RCT	IE 19 GE 19	48%	18–24	20 min cycling with green tree screen vs. blank wall	Total mood disturbance, tension, anger, fatigue, depression, vigor, confusion, self-esteem

GE, green exercise group; IE, indoor exercise group; BUE, build-up exercise group; NE, Non-exercise group.

### Quality assessment of studies

The quality of the included studies was assessed using the Physiotherapy Evidence Database (PEDro) scale,^1^ which evaluates the methodological quality of clinical trials based on 11 items, including randomization, blinding, and statistical analysis. Following the approach used in previous meta-analyses, studies were classified into three quality levels based on PEDro score: high quality (6–10 points), moderate quality (4–5 points), and low quality (≤3 points). Two independent reviewers assessed each study using the PEDro scale, with high inter-rater reliability (*κ* = 0.890; overall agreement rate = 92.3%). Discrepancies were resolved through discussion or consultation with a third reviewer. Additionally, the GRADE system (Grading of Recommendations, Assessment, Development, and Evaluation) was applied to assess the overall certainty of the evidence, taking into account factors such as risk of bias, inconsistency, indirectness, imprecision, and publication bias. GRADE assessments were conducted by two independent reviewers, with discrepancies resolved through discussion.

### Data analyses

Meta-analysis was conducted using R software packages (R version 4.3.0 with R Studio version 2023.06.1 + 524). The standardized mean difference (SMD, Hedges’ g) was used to quantify the differences between the experimental and control groups, based on the mean changes and standard deviations from pre- to post-intervention. A random-effects model was applied to account for expected between-study variability. Effect sizes were classified as trivial (<0.2), small (0.2–0.5), medium (0.5–0.8), and large (>0.8). Heterogeneity was evaluated using I^2^, with values of 25, 50, and 75% indicating low, moderate, and high levels of heterogeneity, respectively. A total of 11 outcome variables were analyzed in the meta-analysis, including overall wellbeing, positive affect (e.g., overall positive affect, calm, vigor), and negative affect (e.g., overall negative affect, stress, depression, anxiety, anger, fatigue, confusion). Additionally, sensitivity analysis was conducted using R packages to assess the robustness of the results.

## Results

### Study characteristics

A total of 3,548 studies were initially identified through databases including PubMed, Web of Science, EBSCOhost, PsycINFO, and the Cochrane Central Register of Controlled Trials. After removing duplicates, 1,596 studies underwent title and abstract screening, followed by full-text screening of 77 studies. Of these, 49 studies met the inclusion criteria. The reasons for excluding the remaining 28 studies are detailed in Supplementary File S2. Additionally, two more studies were identified through reference lists from relevant systematic reviews and meta-analyses, bringing the total to 51 studies included in the meta-analysis. A detailed flow of the screening process is shown in Figure 1.

The characteristics of the studies included in this meta-analysis are summarized in Table 1. A total of 3,092 participants from healthy populations were involved, with ages ranging from 18 to 85 years. Of the 51 studies, 10 focused on male participants, 3 on female participants, and 38 included both genders. The majority of exercise interventions were walking (72.5%), followed by running (13.8%). The studies assessed various outcome measures, including overall wellbeing (*n =* 11), overall positive affect (*n =* 20), calm (*n =* 9), vigor (*n =* 14), overall negative affect (*n =* 18), stress (*n =* 11), depression (*n =* 13), anxiety (*n =* 14), anger (*n =* 12), fatigue (*n =* 10), and confusion (*n =* 6).

### Quality assessment of studies

The results of the PEDro quality assessment are presented in Supplementary File S3. Among the 51 studies, 11 were classified as moderate quality (4–5 points), and 40 as high quality (6–8 points), with a median score of 6 out of 10. Overall, the studies demonstrated moderate to high quality. Funnel plots for all outcomes displayed a generally symmetrical distribution, suggesting no significant publication bias (Supplementary File S4). Furthermore, the GRADE assessment indicated moderate to high certainty in the evidence, reflecting the overall reliability of the findings based on factors such as risk of bias, consistency, imprecision, and publication bias (Supplementary File S5).

### Meta-analysis results

The meta-analysis revealed significant effects of GE on several mental health outcomes compared with IE, BUE, and NE. The main meta-analysis results are presented below and detailed in Table 2. Forest plots illustrating the effects are provided in Supplementary File S6.

**Table 2:** Synthesized results of GE on mental health outcomes compared to NE, IE, and BUE.

Outcome variables	Comparison	k	ES (95% CI)	*P*	I^2^	RW (%)
Overall Wellbeing	GE vs. NE	6	0.46 [0.21, 0.70]	***	0	13.0–22.7
	GE vs. IE	3	0.65 [0.13, 1.16]	*	45.9	30.8–36.8
	GE vs. BUE	4	0.22 [0.00, 0.44]	*	0	15.3–44.9
Overall Positive affect	GE vs. NE	4	1.18 [0.68, 1.69]	***	71	17.2–30.0
	GE vs. IE	9	0.68 [0.34, 1.01]	***	66.6	7.8–12.8
	GE vs. BUE	11	1.01 [0.16, 1.85]	*	92.5	8.6–9.5
Calm	GE vs. NE	3	1.83 [−1.48, 5.14]	0.28	97.7	33.2–33.7
	GE vs. IE	4	0.78 [0.13, 1.43]	*	78.7	20.6–27.5
	GE vs. BUE	5	0.84 [0.22, 1.46]	**	63.6	16.9–26.1
Vigor	GE vs. NE	2	1.18 [−0.25, 2.60]	0.11	75.4	47.4–52.6
	GE vs. IE	5	1.20 [0.23, 2.17]	*	76.7	15.9–23.2
	GE vs. BUE	8	0.59 [0.43, 0.75]	***	0	4.1–27.8
Overall Negative affect	GE vs. NE	4	−0.78 [−1.12, −0.44]	***	43.9	12–33.9
	GE vs. IE	8	−0.31 [−0.62, −0.01]	*	60.4	7.7–16.5
	GE vs. BUE	8	−0.21 [−0.38, −0.05]	*	0	3.6–27.4
Stress	GE vs. NE	3	−0.51 [−0.81, −0.20]	*	0	19.9–58.2
	GE vs. IE	6	−0.34 [−0.56, −0.11]	**	53.5	1.5–41.5
	GE vs. BUE	2	−0.38 [−0.67, −0.09]	**	0	20.9–79.1
Depression	GE vs. NE	4	−0.34 [−0.65, −0.04]	*	0	9.4–57.9
	GE vs. IE	4	−0.88 [−1.55, −0.22]	**	72.7	20.0–31.0
	GE vs. BUE	6	−0.34 [−0.53, −0.14]	***	0	4.6–36.0
Anxiety	GE vs. NE	5	−2.44 [−4.43, −0.45]	*	95.4	18.3–20.7
	GE vs. IE	9	−0.71 [−1.15, −0.28]	**	79.9	8.3–14.0
	GE vs. BUE	4	−0.54 [−0.86, −0.21]	**	0	13.1–57.5
Anger	GE vs. NE	2	−2.42 [−3.61, −1.23]	***	63.2	36.0–64.0
	GE vs. IE	6	−1.37 [−2.65, −0.09]	*	93	15.0–17.5
	GE vs. BUE	6	−0.41 [−0.64, −0.19]	***	0	6.3–28.3
Fatigue	GE vs. NE	3	−3.38 [−7.22, 0.47]	0.09	97.5	33.0–33.8
	GE vs. IE	6	−1.38 [−2.30, −0.45]	**	91.5	14.7–18.0
	GE vs. BUE	4	−0.47 [−0.84, −0.10]	*	34.5	13.0–35.0
Confusion	GE vs. NE	1	0.29 [−0.59, 1.18]	—	—	—
	GE vs. IE	3	−0.64 [−1.10, −0.17]	**	0	23.1–52.3
	GE vs. BUE	3	−0.57 [−0.89, −0.25]	***	0	13.2–56.5

GE, green exercise group; IE, indoor exercise group; BUE, build-up exercise group; NE, Non-exercise group; *P < 0.05, **P < 0.01, ***P < 0.001.

Green Exercise vs. Non-Exercise. Green exercise demonstrated a small but significant improvement in overall wellbeing (SMD = 0.46, 95% CI [0.21, 0.70]). A large effect was observed for overall positive affect (SMD = 1.18 [0.68, 1.69]), while a moderate reduction was found in overall negative affect (SMD = −0.78 [−1.12, −0.44]). For subcomponents of negative affect (e.g., anxiety, depression, stress, anger, fatigue, and confusion), effect sizes ranged from −0.34 to −2.44.

Green Exercise vs. Indoor Exercise. A moderate and significant effect was observed for overall wellbeing (SMD = 0.65 [0.13, 1.16]). Overall positive affect showed a moderate improvement (SMD = 0.68 [0.34, 1.01]), while a small but significant reduction was found in overall negative affect (SMD = −0.31 [−0.62, −0.01]). Subcomponents of positive affect (e.g., vigor and calm) ranged from 0.68 to 0.78, and from −0.31 to −1.38 for negative affect outcomes.

Green Exercise vs. Build-up Exercise. A small yet statistically significant effect was found for overall wellbeing (SMD = 0.22 [0.00, 0.44]). A large effect was observed for overall positive affect (SMD = 1.01 [0.16, 1.85]), and a small but significant reduction was detected in overall negative affect (SMD = −0.21 [−0.38, −0.05]). For subcomponents, effect sizes ranged from 0.59 to 0.84 for positive affect outcomes and from −0.34 to −0.57 for negative affect outcomes.

Some results showed high levels of heterogeneity (> 75%). Sensitivity analysis identified three studies as the primary sources of this heterogeneity (Elsadek et al., 2019; Keenan et al., 2021; Niedermeier et al., 2017). Excluding these studies reduced or eliminated the heterogeneity, while the overall effect sizes remained largely stable. Detailed results of the sensitivity analyses are provided in Supplementary File S7.

## Discussion

This meta-analysis included 51 studies and aimed to assess the effects of green exercise, with a primary focus on comparing green exercise to built-up exercise, indoor exercise, and non-exercise groups. Overall, the findings suggest that green exercise may offer more significant mental health benefits, including improvements in overall wellbeing, positive emotions, and reductions in negative emotions, when compared to built-up exercise, indoor exercise, and non-exercise groups. However, high heterogeneity was observed in several meta-analytic results, which is common in psychological research and may be attributed to differences in scoring methods or participant backgrounds (Wang et al., 2025; Yao et al., 2021a, 2021b). Therefore, caution is warranted when interpreting the pooled results.

This meta-analysis demonstrates that green exercise yields significantly greater mental health benefits compared to both non-exercise and indoor exercise groups. Specifically, when compared to non-exercise, green exercise enhances overall wellbeing, reduces negative emotions (e.g., anxiety, depression, stress, anger, fatigue, and confusion), and fosters positive emotions (e.g., calmness, vigor). These findings underscore the dual influence of physical activity and the restorative effects of natural environments. The mental health benefits of physical activity, such as mood improvement, enhanced self-esteem, and reductions in anxiety and depression, are well-established (Hale et al., 2021; Singh et al., 2023), primarily attributed to physiological responses such as endorphin release and neurochemical changes during exercise (Martín-Rodríguez et al., 2024). Thus, the physical activity aspect of green exercise contributes significantly to the observed mental health improvements. These findings are consistent with those of previous meta-analyses (Coventry et al., 2021; White et al., 2017), which similarly emphasize the mental health benefits of physical activity in natural environments.

In addition to the benefits of exercise itself, green exercise uniquely benefits mental health through its connection to natural settings. While indoor exercise offers physical benefits, it lacks the restorative effects associated with natural environments. Exposure to nature has been shown to support cognitive recovery, reduce stress, and enhance attention, which are vital for emotional regulation (Ohly et al., 2016; Yao et al., 2021a, 2021b). Elements of nature, such as fresh air, natural sounds, and green landscapes, help alleviate mental fatigue, reduce cortisol levels, and induce relaxation, all of which contribute to improved wellbeing (Hedblom et al., 2019; Ikei et al., 2015; Li H. et al., 2022). In contrast, indoor exercise lacks these restorative qualities. The findings of this meta-analysis are consistent with prior meta-analyses and reviews that have highlighted the mental health benefits of nature exposure (Coventry et al., 2021; Firnhaber et al., 2025; Hu et al., 2025). However, this analysis provides greater clarity by differentiating green exercise from indoor exercise and non-exercise controls, and offering a more precise, quantitative assessment of its impact on specific emotional states.

Furthermore, this meta-analysis reveals that green exercise significantly enhances mental health compared to exercise typically conducted in urbanized, built-up environments with limited or no greenery. While both forms of exercise contribute to physical health, green exercise uniquely offers psychological benefits due to its connection to natural environments (Ohly et al., 2016), which are not present in urban settings with limited green spaces. The findings of this meta-analysis align with previous meta-analyses (Wicks et al., 2022), which also highlighted the mental health advantages of nature exposure. A plausible explanation is that urban exercise conditions are often conducted in more built-up environments with limited greenery and greater exposure to urban stressors such as noise and air pollution, whereas indoor exercise lacks direct contact with natural settings, and non-exercise conditions do not involve the combined benefits of physical activity and nature exposure. These differences in environmental and behavioral context may help explain the greater mental health benefits observed for green exercise (Xu et al., 2023). Notably, green exercise appeared to have a stronger effect on positive affect than on negative affect, particularly when compared with built-up exercise. One possible explanation is that green exercise may be more effective in promoting positive emotional states than in alleviating existing negative emotions, which may require longer or more intensive exposure (Yao et al., 2021a, 2021b). In addition, because built-up exercise also involves physical activity in outdoor or real-world settings, the contrast with green exercise may be less pronounced than that with non-exercise or indoor exercise, thereby limiting the additional benefit for negative affect (Wicks et al., 2022). Taken together, these findings suggest that the mental health benefits of exercise may depend not only on physical activity itself but also on the environmental context in which it occurs.

This meta-analysis builds upon a growing body of research exploring the relationship between green exercise and mental health. Previous meta-analyses, such as Wicks et al. (2022), Brito et al. (2022), Hu et al. (2025), have consistently demonstrated that green exercise has a small to moderate positive impact on mental health. Broader meta-analyses on nature exposure and wellbeing, including Twohig-Bennett and Jones (2018), Liu et al. (2023), also show that nature exposure improves mental health across various population. Our findings are largely consistent with these results, further confirming the mental health benefits of green exercise. However, this review contributes to the existing literature in several important ways. Unlike previous reviews that either focus broadly on nature exposure or face methodological limitations such as limited statistical power, imprecise definitions of green exercise, and inconsistent comparator groups, this review focuses specifically on the impact of green exercise interventions. It systematically compares their effects with those of non-exercise, indoor exercise, and built-up urban environments. Furthermore, by providing a detailed analysis of the effects across different comparison groups, this review highlights where green exercise may be most effective (e.g., compared to built-up exercise), and identifies areas where its effects may be less distinguishable (e.g., compared to indoor or urban built-up exercise).

### Limitations

This meta-analysis provides valuable insights into the effects of green exercise on mental health but has several limitations that need to be considered. First, most of the included studies were based on short-term interventions, with follow-up durations generally under three months, limiting the ability to assess the long-term sustainability of green exercise’s mental health benefits. Second, the majority of included studies examined green exercise in the form of walking, with limited evidence on other types of physical activity (e.g., running, cycling) conducted in green spaces. The small number of studies for other exercise types prevented subgroup analyses by activity mode. Consequently, the findings primarily reflect the effects of low-intensity walking in natural environments, and caution is warranted when generalizing these results to other forms of green exercise that may involve different intensities or modes of activity. Third, high heterogeneity was observed across several meta-analyses, likely due to variations in study designs, physical activity types, and environmental conditions, which may have influenced the psychological outcomes. Additionally, many studies had small sample sizes and methodological limitations, such as inadequate reporting of randomization procedures and limited blinding, which increased the risk of bias. Finally, the limited number of studies included prevented subgroup analyses, and future research with larger, more standardized studies is needed to better understand the long-term effects and specific conditions under which green exercise is most effective.

## Conclusion

This systematic review and meta-analysis confirm that green exercise offers significant mental health benefits, enhancing overall wellbeing, positive affect, and reducing negative emotions. These findings highlight the potential for integrating green exercise into public health initiatives, urban planning, and wellness programs to promote mental wellbeing across diverse populations. Further research is needed to explore the long-term effects of green exercise and to identify the specific conditions under which its benefits are maximized.

## Data Availability

The original contributions presented in the study are included in the article/Supplementary material, further inquiries can be directed to the corresponding author.

## Footnotes

1
https://www.pedro.org.au
