# Supplementary material for: Effects of green exercise on mental health: a systematic review and meta-analysis
Source: Front Psychol. 2026 Apr 14;17:1802759. doi: 10.3389/fpsyg.2026.1802759 (PMC13121117; doi:10.3389/fpsyg.2026.1802759)
Supplement: Supplementary file 1 [file Data_Sheet_1.pdf]

S1 File. Search Alert (7.11.2025)

Pubmed (406)
((green[Title] OR outside[Title] OR outdoor[Title] OR nature[Title] OR forest[Title] OR park[Title] OR garden[Title] OR landscape[Title] OR plant[Title] OR wood[Title] OR flower[Title]) AND (exercise[Title] OR physical activity[Title] OR physical fit[Title] OR walking[Title] OR hiking[Title] OR cycling[Title] OR jogging[Title] OR running[Title] OR aerobics[Title])) AND (Psychology[Title/Abstract] OR emotion[Title/Abstract] OR mood[Title/Abstract] OR affect[Title/Abstract] OR mental[Title/Abstract] OR well-being[Title/Abstract] OR depression[Title/Abstract] OR anxiety[Title/Abstract] OR stress[Title/Abstract]) AND (elderly[Title/Abstract] OR older adults[Title/Abstract])

Web of Science (1458)
[Title] (green OR outside OR outdoor OR nature OR forest OR park OR garden OR landscape OR plant OR wood OR flower) AND [Title] (exercise OR physical activity OR physical fit OR walking OR hiking OR cycling OR jogging OR running OR aerobics) AND [Abstract] (Psychology OR emotion OR mood OR affect OR mental OR well-being OR depression OR anxiety OR stress) AND [Abstract] (elderly OR older adults)

EBSCOhost (1068)
TI (green OR outside OR outdoor OR nature OR forest OR park OR garden OR landscape OR plant OR wood OR flower) AND TI (exercise OR physical activity OR physical fit OR walking OR hiking OR cycling OR jogging OR running OR aerobics) AND AB (Psychology OR emotion OR mood OR affect OR mental OR well-being OR depression OR anxiety OR stress) AND AB (elderly OR older adults)

PsycINFO (447)
Title: green OR Title: outside OR Title: outdoor OR Title: nature OR Title: forest OR Title: park OR Title: garden OR Title: landscape OR Title: plant OR Title: wood OR Title: flower AND Abstract: exercise OR Abstract: physical activity OR Abstract: physical fit OR Abstract: walking OR Abstract: hiking OR Abstract: cycling OR Abstract: jogging OR Abstract: running OR Abstract: aerobics AND Abstract: Psychology OR Abstract: emotion OR Abstract: mood OR Abstract: affect OR Abstract: mental OR Abstract: well-being OR Abstract: depression OR Abstract: anxiety OR Abstract: stress AND Abstract: elderly OR Abstract: older adults

Cochrane Central Register of Controlled Trials (169)
[Title Abstract Keyword] (green OR outside OR outdoor OR nature OR forest OR park OR garden OR landscape OR plant OR wood OR flower) AND [Title Abstract Keyword (exercise OR physical activity OR physical fit OR walking OR hiking OR cycling OR jogging OR running OR aerobics) AND [Title Abstract Keyword] (Psychology OR emotion OR mood OR affect OR mental OR well-being OR depression OR anxiety OR stress) AND [Title Abstract Keyword] (elderly OR older adults)

S2 File. References for Excluded Studies

**Article**	**Reason for exclusion**
Brito, H., Lopes, H., de Carvalho, M. V., Carrilho, D., Carvalho, A., & Araújo, D. (2024). The effects of nature-based vs. indoor settings on the adaptability, performance and affect of calisthenics exercisers. A registered report. Psychology of Sport and Exercise, 73, 102626.	The outdoor intervention group did not include green environments.
Crust, L., Henderson, H., & Middleton, G. (2013). The acute effects of urban green and countryside walking on psychological health: a field-based study of green exercise.	Full text is not available.
Duncan, M. J., Clarke, N. D., Birch, S. L., Tallis, J., Hankey, J., Bryant, E., & Eyre, E. L. (2014). The effect of green exercise on blood pressure, heart rate and mood state in primary school children. International journal of environmental research and public health, 11(4), 3678-3688.	Study participants under 18 years old.
Focht, B. C. (2009). Brief walks in outdoor and laboratory environments: effects on affective responses, enjoyment, and intentions to walk for exercise. Research quarterly for exercise and sport, 80(3), 611-620.	The outdoor intervention group did not include green environments.
Glover, N., & Polley, S. (2019). Going green: the effectiveness of a 40-day green exercise intervention for insufficiently active adults. Sports, 7(6), 142.	No non-green counterpart.
Han, K.-T. (2021). Effects of three levels of green exercise, physical and social environments, personality traits, physical activity, and engagement with nature on emotions and attention. Sustainability, 13(5), 2686.	No non-green counterpart.
Iwata, Y., Dhubháin, Á. N., Brophy, J., Roddy, D., Burke, C., & Murphy, B. (2016). Benefits of group walking in forests for people with significant mental ill-health. Ecopsychology, 8(1), 16-26.	No non-green counterpart.
Janeczko, E., Bielinis, E., Wójcik, R., Woźnicka, M., Kędziora, W., Łukowski, A., Elsadek, M., Szyc, K., & Janeczko, K. (2020). When urban environment is restorative: The effect of walking in suburbs and forests on psychological and physiological relaxation of young Polish adults. Forests, 11(5), 591.	No non-green counterpart.
Korpela, K. M., Stengård, E., & Jussila, P. (2016). Nature walks as a part of therapeutic intervention for depression. Ecopsychology, 8(1), 8-15.	No non-green counterpart.
Koselka, E. P., Weidner, L. C., Minasov, A., Berman, M. G., Leonard, W. R., Santoso, M. V., de Brito, J. N., Pope, Z. C., Pereira, M. A., & Horton, T. H. (2019). Walking green: developing an evidence base for nature prescriptions. International journal of environmental research and public health, 16(22), 4338.	No non-green counterpart.
Kwon S, Lee K, Ahn J. 2018. Affective changes in different physical environments and the effects of "Green Exercise" A mixed method approach. International Journal of Sport Psychology. May-Jun; 49:201-223.	Full text is not available.
Lawton E, Brymer E, Clough P, Denovan A. 2017. The Relationship between the Physical Activity Environment, Nature Relatedness, Anxiety, and the	Cross-sectional design.

Psychological Well-being Benefits of Regular Exercisers. Frontiers in psychology. Jun 26;8.	
Lopez-Pousa S, Bassets Pages G, Monserrat-Vila S, de Gracia Blanco M, Hidalgo Colome J, Garre-Olmo J. 2015. Sense of Well-Being in Patients with Fibromyalgia: Aerobic Exercise Program in a Mature Forest-A Pilot Study. Evidence-Based Complementary and Alternative Medicine. 2015;2015.	Data is not available.
Mackay, G. J., & Neill, J. T. (2010). The effect of "green exercise" on state anxiety and the role of exercise duration, intensity, and greenness: A quasi-experimental study. Psychology of Sport and Exercise, 11(3), 238-245.	No non-green counterpart.
Marselle, M. R., Warber, S. L., & Irvine, K. N. (2019). Growing resilience through interaction with nature: Can group walks in nature buffer the effects of stressful life events on mental health? International journal of environmental research and public health, 16(6), 986.	Cross-sectional design.
McCaffrey, R., & Liehr, P. (2016). The effect of reflective garden walking on adults with increased levels of psychological stress. Journal of Holistic Nursing, 34(2), 177-184.	No non-green counterpart.
McEwan K, Giles D, Clarke FJ, Kotera Y, Evans G, Terebenina O, Minou L, Teeling C, Basran J, Wood W, et al. 2021. A Pragmatic Controlled Trial of Forest Bathing Compared with Compassionate Mind Training in the UK: Impacts on Self-Reported Wellbeing and Heart Rate Variability. Sustainability. Feb;13.	Inappropriate study design.
Müller-Riemenschneider, F., Petrunoff, N., Yao, J., Ng, A., Sia, A., Ramiah, A., Wong, M., Han, J., Tai, B. C., & Uijtdewilligen, L. (2020). Effectiveness of prescribing physical activity in parks to improve health and wellbeing-the park prescription randomized controlled trial. International Journal of Behavioral Nutrition and Physical Activity, 17(1), 42.	Data is not available.
Muro, A., Mateo, C., Parrado, E., Subirana-Malaret, M., Moya, M., Garriga, A., Canals, J., Chamarro, A., & Sanz, A. (2023). Forest bathing and hiking benefits for mental health during the COVID-19 pandemic in Mediterranean regions. European Journal of Forest Research, 142(2), 415-426.	No non-green counterpart.
Noushad, S., Ansari, B., & Ahmed, S. (2022). Effect of nature-based physical activity on post-traumatic growth among healthcare providers with post-traumatic stress. Stress and health, 38(4), 813-826.	The research subjects were not healthy.
Perkins, S., Searight, H. R., & Ratwik, S. (2011). Walking in a natural winter setting to relieve attention fatigue: A pilot study. Psychology, 2(8), 777.	No non-green counterpart.
Rantanen, T., Äyräväinen, I., Eronen, J., Lyyra, T., Törmäkangas, T., Vaarama, M., & Rantakokko, M. (2015). The effect of an outdoor activities' intervention delivered by older volunteers on the quality of life of older people with severe mobility limitations: a randomized controlled trial. Aging clinical and experimental research, 27(2), 161-169.	The outdoor intervention group did not include green environments.

Rogerson, M., Brown, D. K., Sandercock, G., Wooller, J.-J., & Barton, J. (2016). A comparison of four typical green exercise environments and prediction of psychological health outcomes. Perspectives in public health, 136(3), 171-180.	No non-green counterpart.
Rogerson, M., Colbeck, I., Bragg, R., Dosumu, A., & Griffin, M. (2020). Affective outcomes of group versus lone green exercise participation. International journal of environmental research and public health, 17(2), 624.	Inappropriate study design.
Ryu, J., Jung, J. H., Kim, J., Kim, C.-H., Lee, H.-B., Kim, D.-H., Lee, S.-K., Shin, J.-H., & Roh, D. (2020). Outdoor cycling improves clinical symptoms, cognition and objectively measured physical activity in patients with schizophrenia: A randomized controlled trial. Journal of psychiatric research, 120, 144-153.	Inappropriate control group.
Shrestha T, Di Blasi Z, Cassarino M. 2021. Natural or Urban Campus Walks and Vitality in University Students: Exploratory Qualitative Findings from a Pilot Randomised Controlled Study. International journal of environmental research and public health. Feb;18.	Data is not available.
Wade, L., Lubans, D. R., Smith, J. J., & Duncan, M. J. (2020). The impact of exercise environments on adolescents' cognitive and psychological outcomes: A randomised controlled trial. Psychology of Sport and Exercise, 49, 101707.	Study participants under 18 years old.
Wang, X., Zhou, Q., Zhang, M., & Zhang, Q. (2021). Exercise in the park or gym? The physiological and mental responses of obese people walking in different settings at different speeds: a parallel group randomized trial. Frontiers in Psychology, 12, 728826.	Data is not available.

S3 File. Study Quality Assessment

Table S1. Physiotherapy Evidence Database (PEDro) scale ratings

**References**	**Items***											**Total (out of 10)**
	**1**	**2**	**3**	**4**	**5**	**6**	**7**	**8**	**9**	**10**	**11**	
Anandh 2021	1	0	0	1	0	0	0	1	1	1	1	5
Anzman-Frasca 2023	1	1	0	1	1	0	0	1	1	1	1	7
Bang 2017	1	0	0	1	0	0	0	1	1	1	1	5
Barton 2012	1	0	0	1	0	0	0	1	1	1	1	5
Berman 2012	1	1	0	1	1	0	0	1	1	1	1	7
Bodin 2003	1	1	0	1	1	0	0	1	1	1	1	7
Bramwell 2023	1	1	1	1	0	0	0	1	1	1	1	7
Brown 2014	1	1	1	1	0	0	0	1	1	1	1	7
Byrka 2018	1	1	0	1	1	0	0	1	1	1	1	7
Calogiuri 2018	1	0	0	1	1	0	0	1	1	1	1	6
Carter 2022	1	0	0	1	1	0	0	1	1	1	1	6
Crossan 2019	1	0	0	1	1	0	0	1	1	1	1	6
de Brito 2019	1	0	0	1	1	0	0	1	1	1	1	6
Dickmeyer 2025	1	1	1	1	0	0	0	1	1	1	1	7
Elsadek 2019	1	0	0	1	1	0	0	1	1	1	1	6
Fuegen 2018	1	1	0	1	1	0	0	1	1	1	1	7
Geniole 2016	1	0	0	1	1	0	0	1	1	1	1	6
Gidlow 2016	1	1	1	1	1	0	0	1	1	1	1	8
Harte 1995	1	0	0	0	0	0	0	1	1	1	1	4
Hartig 2003	1	1	0	0	1	0	0	1	1	1	1	6
Hvid 2025	1	1	1	1	1	0	0	1	1	1	1	8
Jang 2017	1	1	0	0	0	0	0	1	1	1	1	5
Johansson 2011	1	0	0	1	0	0	0	1	1	1	1	5
Keenan 2021	1	1	0	0	0	0	0	1	1	1	1	5
Kerr 2006	1	0	0	1	1	0	0	1	1	1	1	6
Kinnafick 2014	1	0	0	1	1	0	0	1	1	1	1	6
Klaperski 2019	1	0	0	1	0	0	0	1	1	1	1	5
Lee 2014	1	1	0	1	1	0	0	1	1	1	1	7
Legrand 2022	1	1	0	1	1	0	0	1	1	1	1	7
Levinger 2023	1	1	1	1	1	0	0	1	1	1	1	8
Li 2021	1	1	0	1	0	0	0	1	1	1	1	6
Ma 2023	1	1	1	1	0	0	0	1	1	1	1	7
Mao 2012	1	1	0	0	1	0	0	1	1	1	1	6
Marselle 2013	1	0	0	0	0	0	0	1	1	1	1	4
Sales 2017	1	1	1	1	0	0	0	1	1	1	1	7
Menzel 2020	1	0	0	1	1	0	0	1	1	1	1	6
Niedermeier 2017	1	1	0	1	1	0	0	1	1	1	1	7
Nisbet 2011	1	1	0	0	1	0	0	1	1	1	1	6
Ojala 2019	1	0	0	1	1	0	0	1	1	1	1	6
Olafsdottir 2018	1	1	0	1	0	0	0	1	1	1	1	6
Park 2007	1	1	0	1	1	0	0	1	1	1	1	7
Park 2009	1	1	0	1	1	0	0	1	1	1	1	7
Park 2024	1	1	0	1	0	0	0	1	1	1	1	6
Shin 2013	1	1	0	0	0	0	0	1	1	1	1	5

References	Items*											Total (out of 10)
	**1**	**2**	**3**	**4**	**5**	**6**	**7**	**8**	**9**	**10**	**11**	
Takayama 2014	1	1	0	1	1	0	0	1	1	1	1	7
Teas 2007	1	0	0	1	1	0	0	1	1	1	1	6
Trammell 2020	1	0	0	1	1	0	0	1	1	1	1	6
Turner 2017	1	0	0	1	1	0	0	1	1	1	1	6
Tyrväinen 2014	1	0	0	1	1	0	0	0	1	1	1	5
Watkins-Martin 2022	1	1	1	0	0	0	0	0	1	1	1	5
Zhang 2023	1	1	0	1	0	0	0	1	1	1	1	6
												Median score = 6

Note: * Detailed explanations for PEDro scale items are available at https://pedro.org.au/English/resources/pedro-scale/ (access for this review: November 7, 2025).

S4 File. Funnel plots

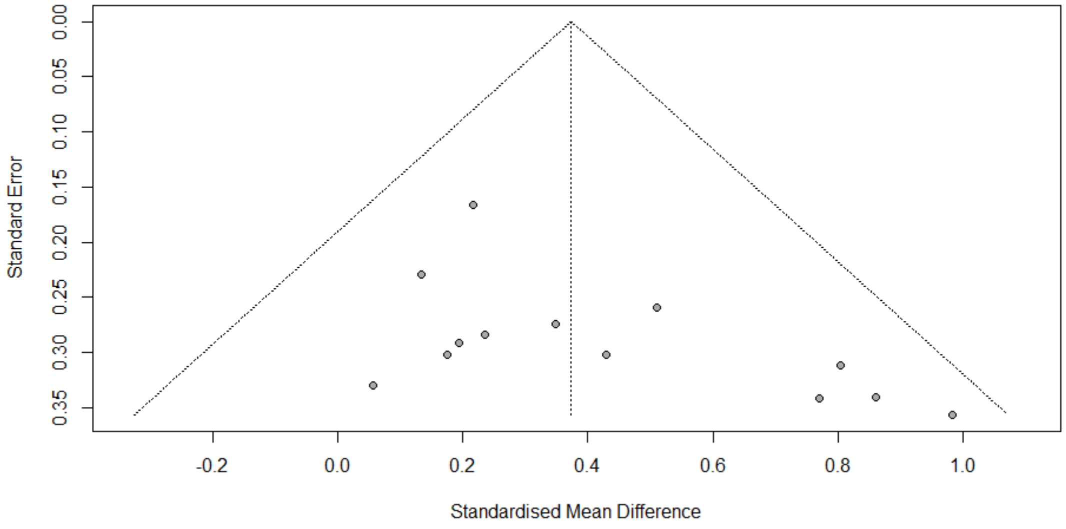

Fig S1. Funnel plot for well-being

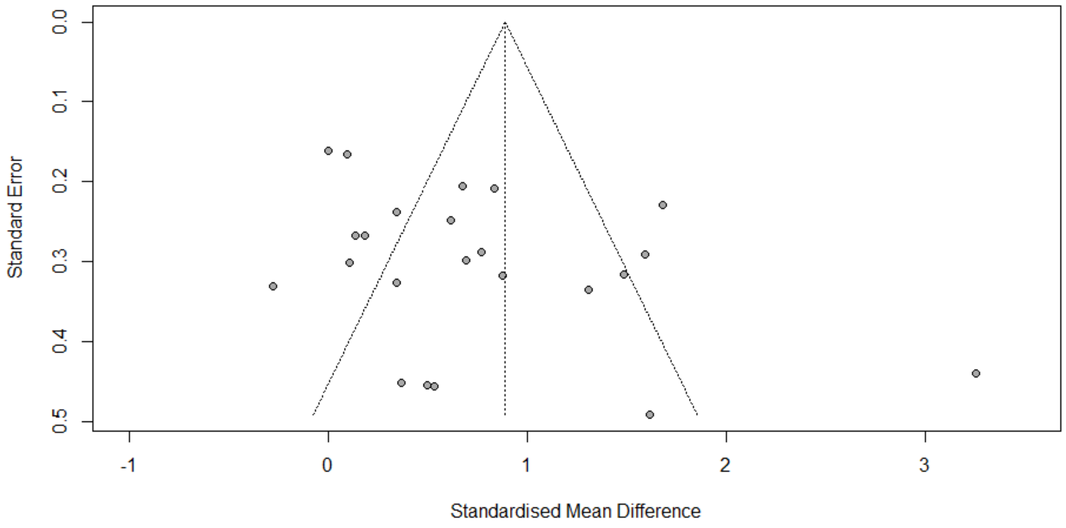

Fig S2. Funnel plot for positive affect

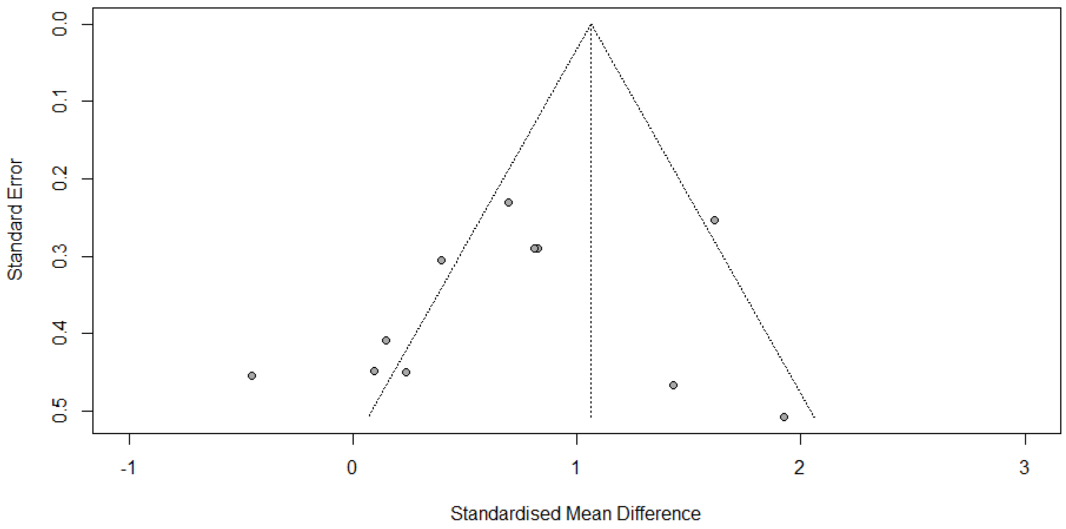

Fig S3. Funnel plot for calm

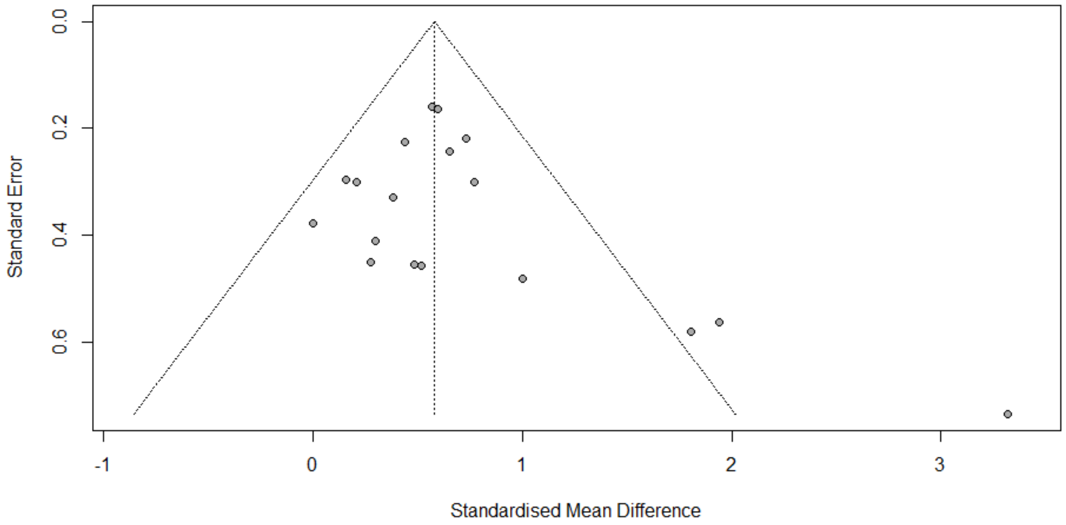

Fig S4. Funnel plot for vigor

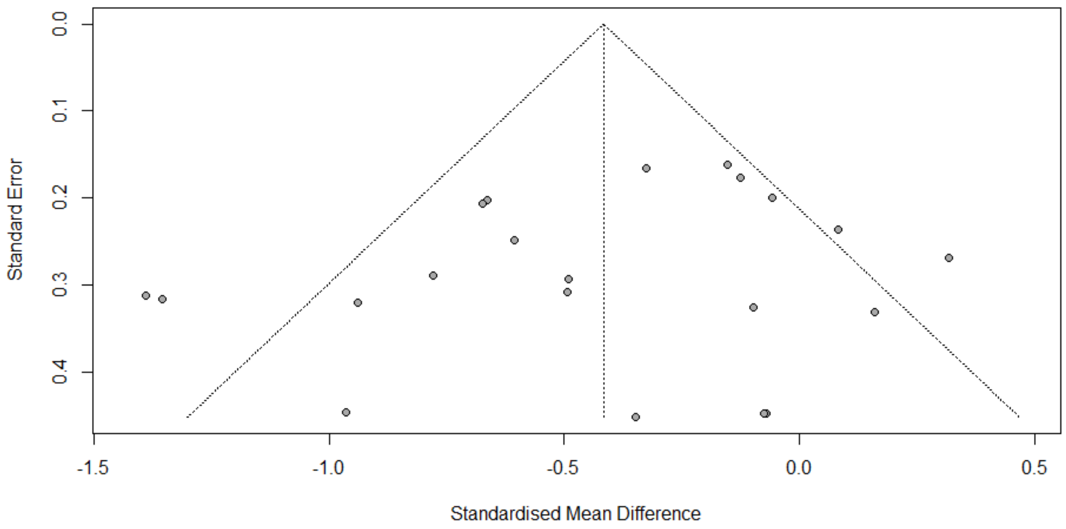

Fig S5. Funnel plot for negative affect

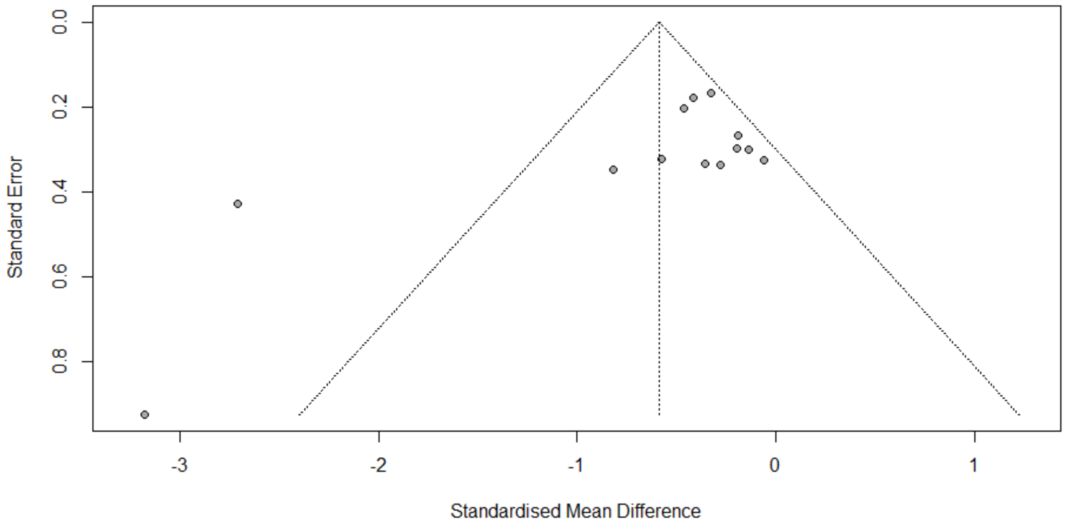

Fig S6. Funnel plot for stress

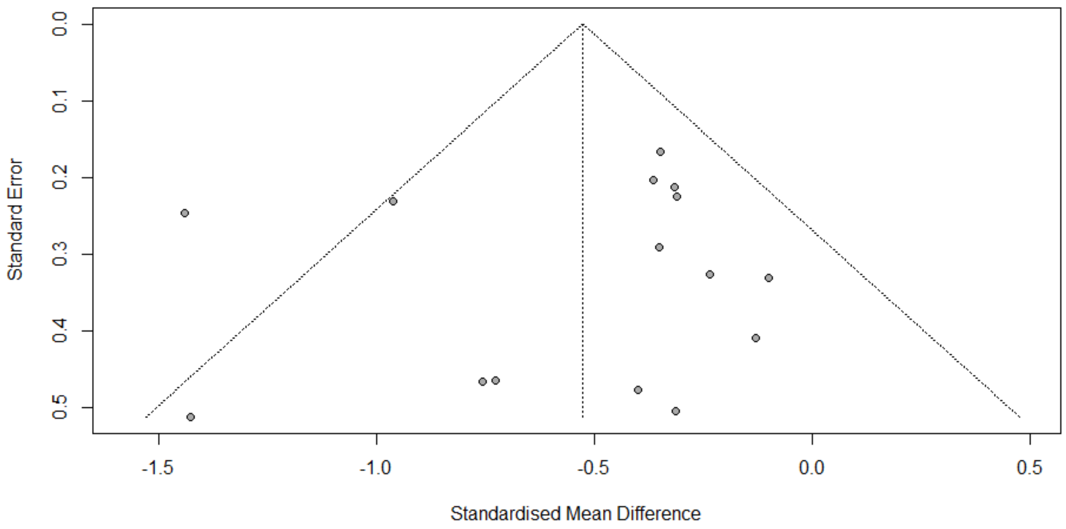

Fig S7. Funnel plot for depression

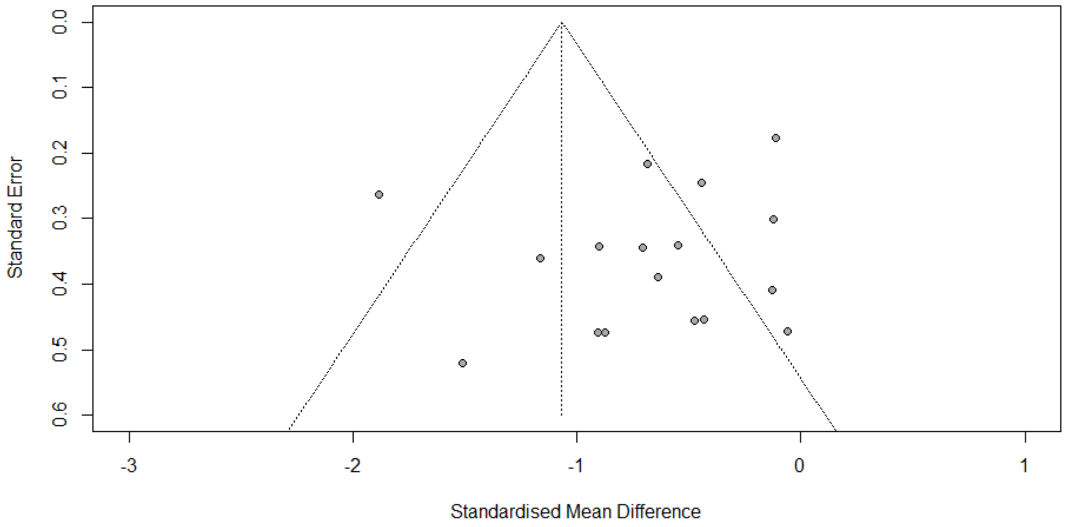

Fig S8. Funnel plot for anxiety

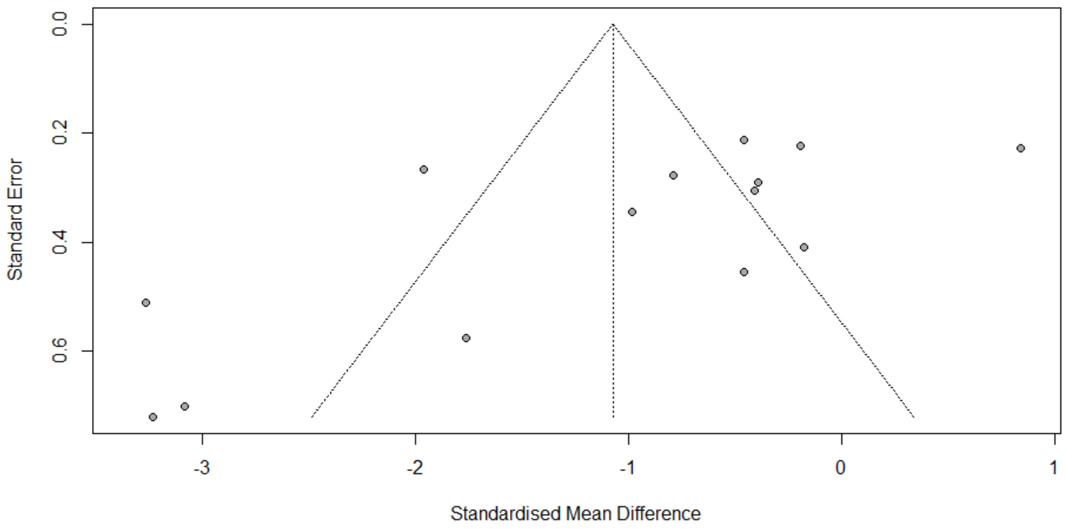

Fig S9. Funnel plot for anger

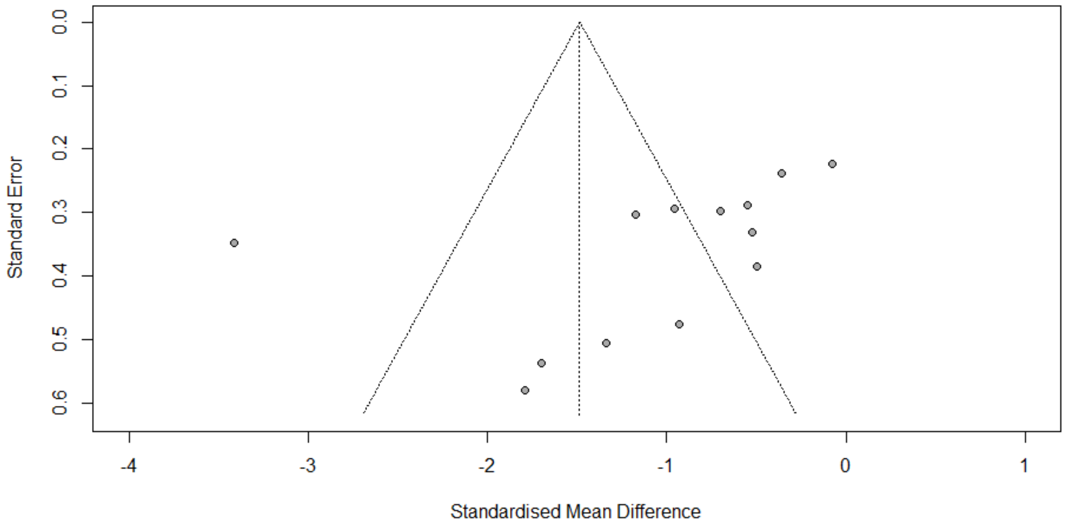

Fig S10. Funnel plot for fatigue

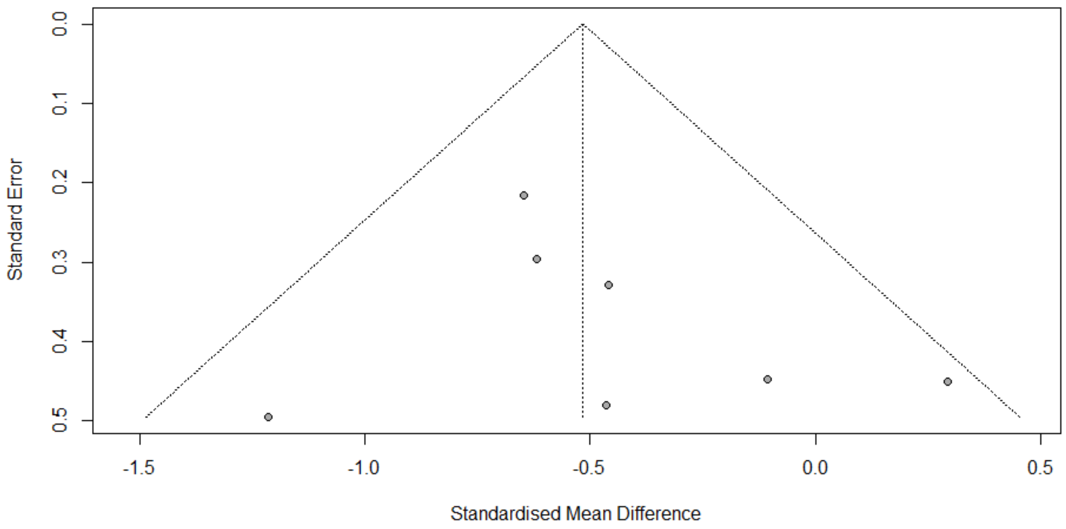

Fig S11. Funnel plot for confusion

S5 File. GRADE evidence profile

Author(s): Xinyi Liu, Delong Dong, Xiaolin Wang
Date: 2025-11-19
Question: Should green exercise be used for mental health?
Settings: exercise
Bibliography: Anandh et al., 2021; Anzman-Frasca et al., 2023; Bang et al., 2017; Barton et al., 2012; Berman et al., 2012; Bodin & Hartig, 2003; Bramwell et al., 2023; Brown et al., 2014; Byrka & Ryczko, 2018; Calogiuri et al., 2018; Carter et al., 2022; Crossan & Salmoni, 2021; de Brito et al., 2019; Dickmeyer et al., 2025; Elsadek et al., 2019; Fuegen & Breitenbecher, 2018; Geniole et al., 2016; Gidlow et al., 2016; Harte & Eifert, 1995; Hartig et al., 2003; Hvid et al., 2025; Jang & So, 2017; Johansson et al., 2011; Keenan et al., 2021; Kerr et al., 2006; Kinnafick & Thøgersen-Ntoumani, 2014; Klaperski et al., 2019; Lee et al., 2014; Legrand et al., 2022; Levinger et al., 2023; Li et al., 2021; Ma et al., 2023; Mao et al., 2012; Marselle et al., 2013; Menzel et al., 2020; Niedermeier et al., 2017; Nisbet & Zelenski, 2011; Ojala et al., 2019; Olafsdottir et al., 2020; Park et al., 2007; Park et al., 2009; Park et al., 2024; Sales et al., 2017; Shin et al., 2013; Takayama et al., 2014; Teas et al., 2007; Trammell & Aguilar, 2021; Turner & Stevinson, 2017; Tyrväinen et al., 2014; Watkins-Martin et al., 2022; Zhang et al., 2023

Quality assessment							No of patients		Effect		Quality	Importance
No of studies	Design	Risk of bias	Inconsistency	Indirectness	Imprecision	Other considerations	Green exercise	Control	Relative (95% CI)	Absolute		
**Overall Well-being (Better indicated by higher values)**												
11	randomised trials	serious[1]	no serious inconsistency	no serious indirectness	no serious imprecision	none	476	270	-	SMD 0.44 higher (0.17 to 0.77 higher)	⊕⊕⊕O MODERATE	CRITICAL
**Overall Positive affect (Better indicated by higher values)**												
20	randomised trials	serious[2]	no serious inconsistency	no serious indirectness	no serious imprecision	strong association	781	616	-	SMD 0.96 higher (0.39 to 1.52 higher)	⊕⊕⊕⊕ HIGH	CRITICAL
**Overall Negative affect (Better indicated by higher values)**												
18	randomised trials	serious[3]	no serious inconsistency	no serious indirectness	no serious imprecision	none	764	549	-	SMD 0.43 lower (0.71 to 0.17 lower)	⊕⊕⊕O MODERATE	CRITICAL
**Calm (Better indicated by higher values)**												
9	randomised trials	serious[4]	no serious inconsistency	no serious indirectness	serious[5]	strong association	168	168	-	SMD 1.15 higher (0.38 lower to 2.68 higher)	⊕⊕⊕O MODERATE	IMPORTANT
**Vigor (Better indicated by higher values)**												
14	randomised trials	serious[6]	no serious inconsistency	no serious indirectness	no serious imprecision	strong association	439	430	-	SMD 0.99 higher (0.14 to 1.84 higher)	⊕⊕⊕⊕ HIGH	IMPORTANT
**Stress (Better indicated by higher values)**												
11	randomised trials	serious[7]	no serious inconsistency	no serious indirectness	no serious imprecision	none	516	287	-	SMD 0.41 lower (0.68 to 0.13 lower)	⊕⊕⊕O MODERATE	IMPORTANT
**Depression (Better indicated by higher values)**												
13	randomised trials	serious[7]	no serious inconsistency	no serious indirectness	no serious imprecision	none	506	328	-	SMD 0.52 lower (0.91 to 0.13 lower)	⊕⊕⊕O MODERATE	IMPORTANT
**Anxiety (Better indicated by higher values)**												
14	randomised trials	serious[8]	no serious inconsistency	no serious indirectness	no serious imprecision	strong association	335	285	-	SMD 1.23 lower (2.15 to 0.31 lower)	⊕⊕⊕⊕ HIGH	IMPORTANT
**Anger (Better indicated by higher values)**												
12	randomised trials	serious[9]	no serious inconsistency	no serious indirectness	no serious imprecision	strong association	280	280	-	SMD 1.40 lower (2.30 to 0.50 lower)	⊕⊕⊕⊕ HIGH	IMPORTANT
**Fatigue (Better indicated by higher values)**												
10	randomised trials	serious[8]	no serious inconsistency	no serious indirectness	serious[10]	strong association	255	255	-	SMD 1.74 lower (3.45 to 0.34 lower)	⊕⊕⊕O MODERATE	IMPORTANT
**Confusion (Better indicated by higher values)**												
6	randomised trials	serious[11]	no serious inconsistency	no serious indirectness	no serious imprecision	none	117	117	-	SMD 0.31 lower (0.86 lower to 0.25 higher)	⊕⊕⊕O MODERATE	IMPORTANT

[1] 8 studies did not mention whether group allocation was concealed, and most studies did not report blinding information
[2] 19 studies did not mention whether group allocation was concealed, and most studies did not report blinding information
[3] 17 studies did not mention whether group allocation was concealed, and most studies did not report blinding information
[4] Most studies lacked relevant information regarding allocation concealment and blinding.
[5] The confidence interval is wide
[6] Most studies lacked relevant information regarding allocation concealment and blinding.
[7] 10 studies did not mention whether group allocation was concealed, and most studies did not report blinding information
[8] 9 studies did not mention whether group allocation was concealed, and most studies did not report blinding information
[9] 11 studies did not mention whether group allocation was concealed, and most studies did not report blinding information
[10] The confidence interval is wide
[11] 6 studies did not mention whether group allocation was concealed, and most studies did not report blinding information

Fig S12. GRADE evidence profile

S6 File. Forest plots

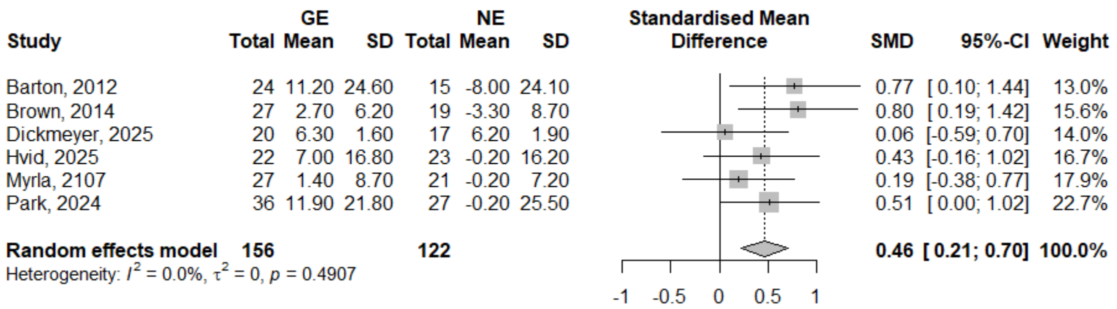

Fig S13. Forest plot of well-being: GE vs. NE

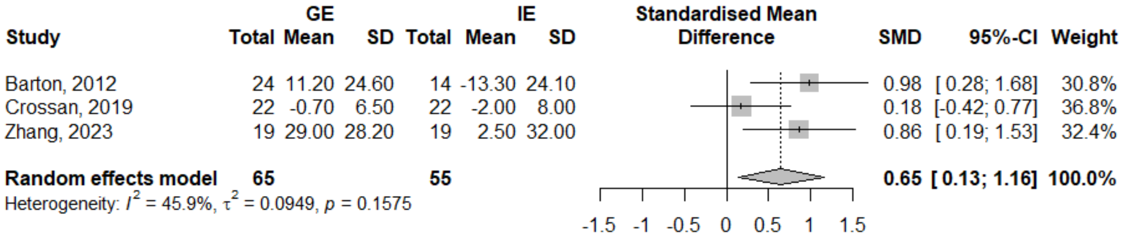

Fig S14. Forest plot of well-being: GE vs. IE

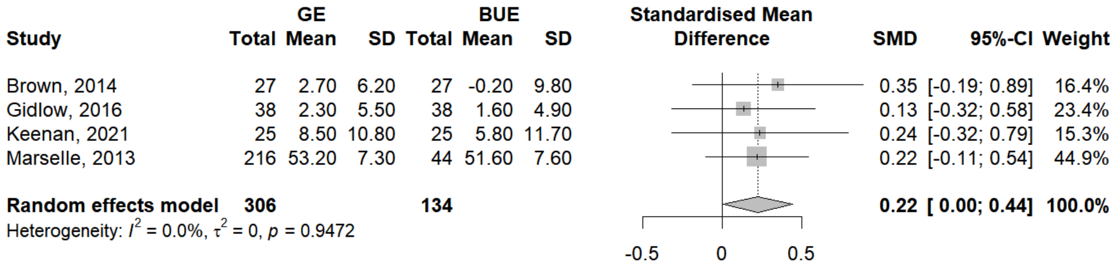

Fig S15. Forest plot of well-being: GE vs. BUE

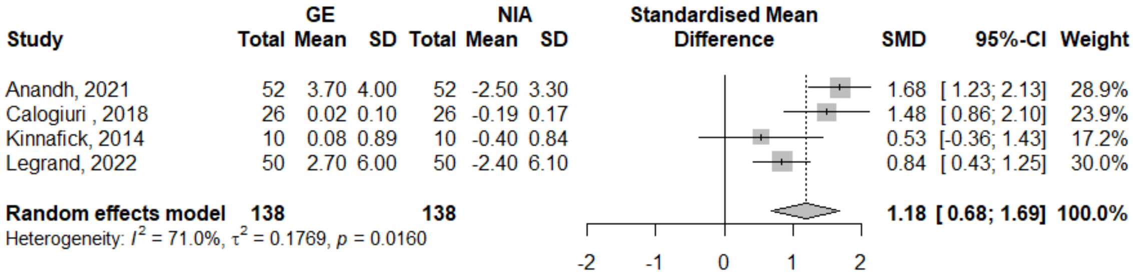

Fig S16. Forest plot of positive affect: GE vs. NIA

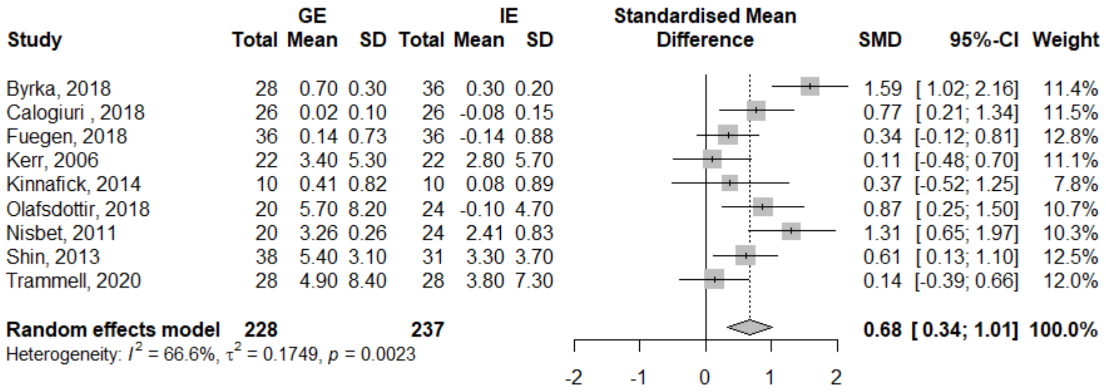

Fig S17. Forest plot of positive affect: GE vs. IE

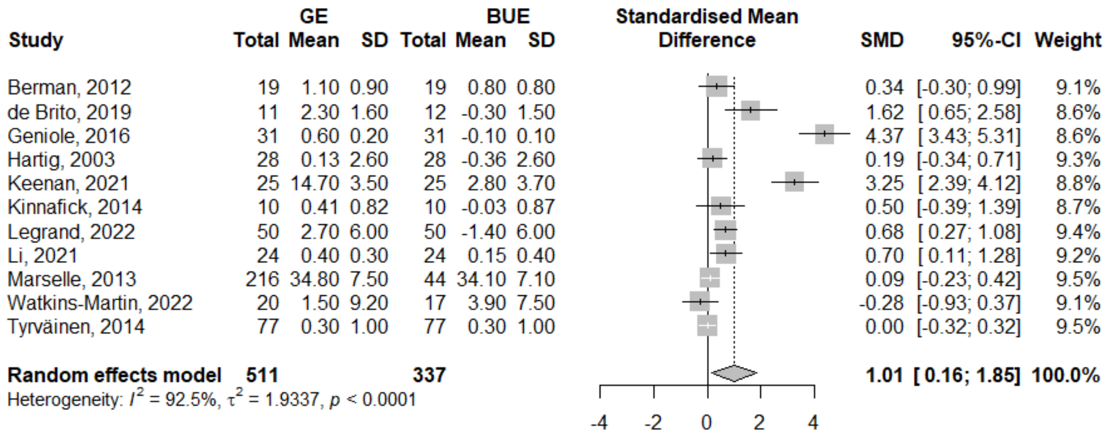

Fig S18. Forest plot of positive affect: GE vs. BUE

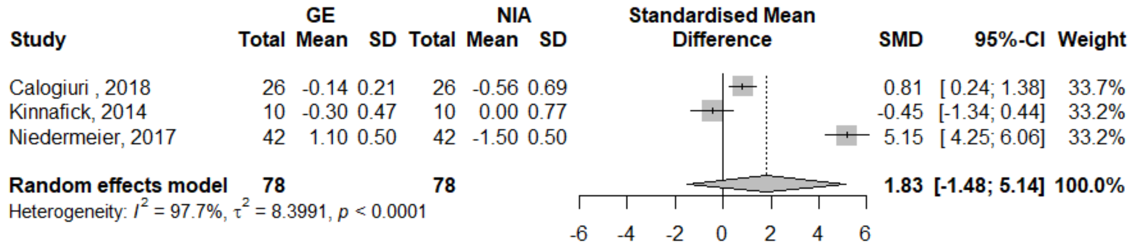

Fig S19. Forest plot of calm: GE vs. NIA

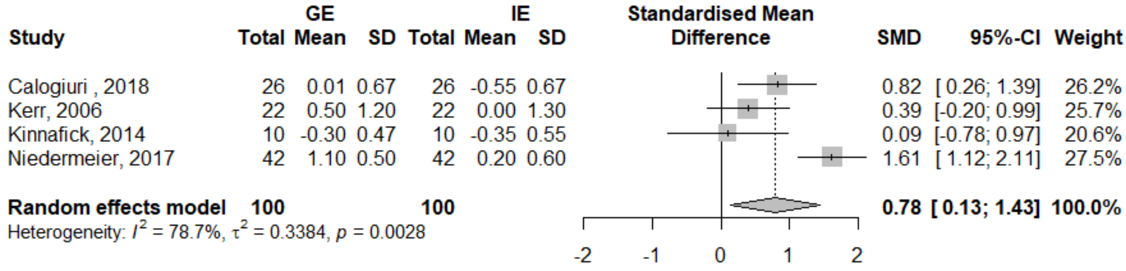

Fig S20. Forest plot of calm: GE vs. IE

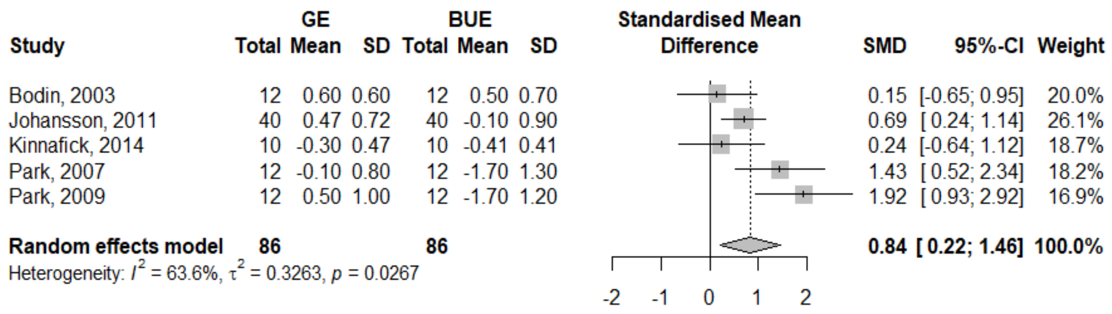

Fig S21. Forest plot of calm: GE vs. BUE

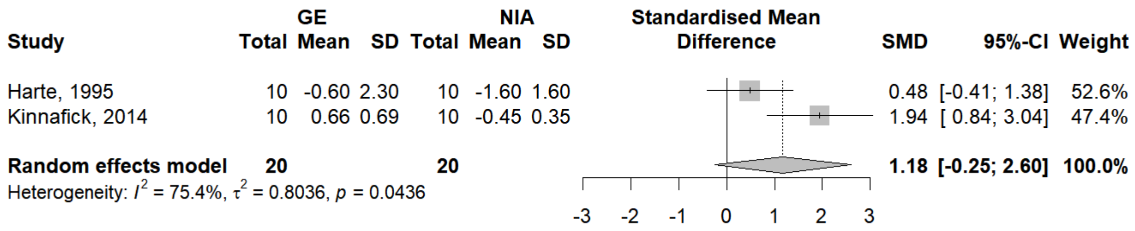

Fig S22. Forest plot of vigor: GE vs. NIA

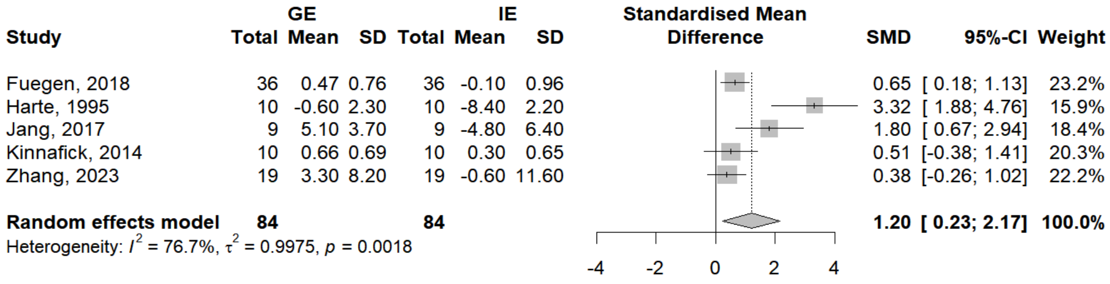

Fig S23. Forest plot of vigor: GE vs. IE

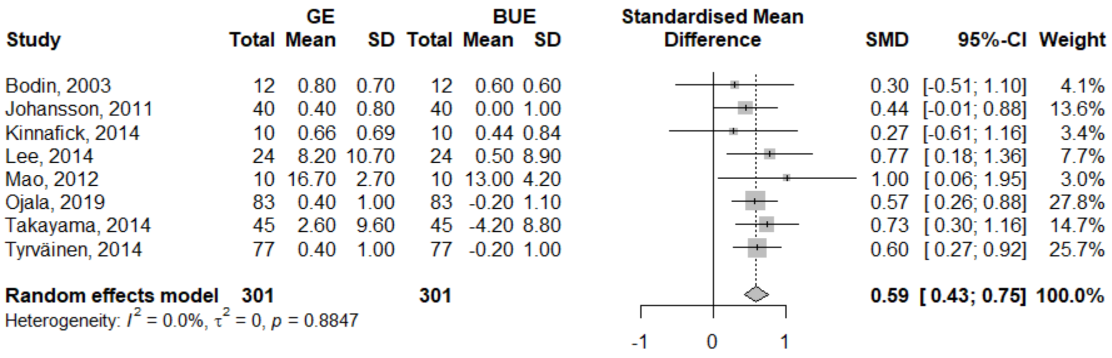

Fig S24. Forest plot of vigor: GE vs. BUE

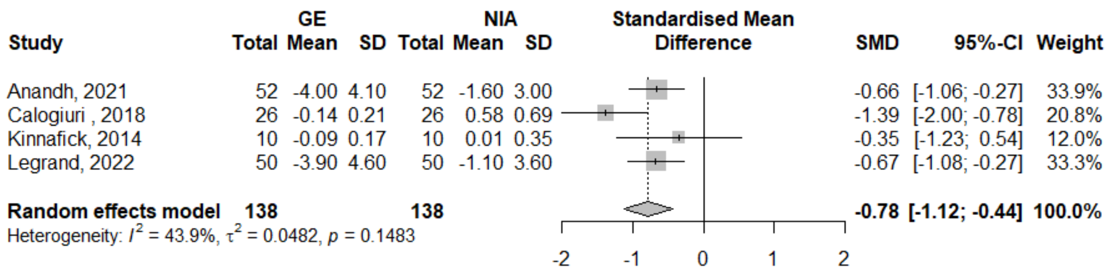

Fig S25. Forest plot of negitive affect: GE vs. NIA

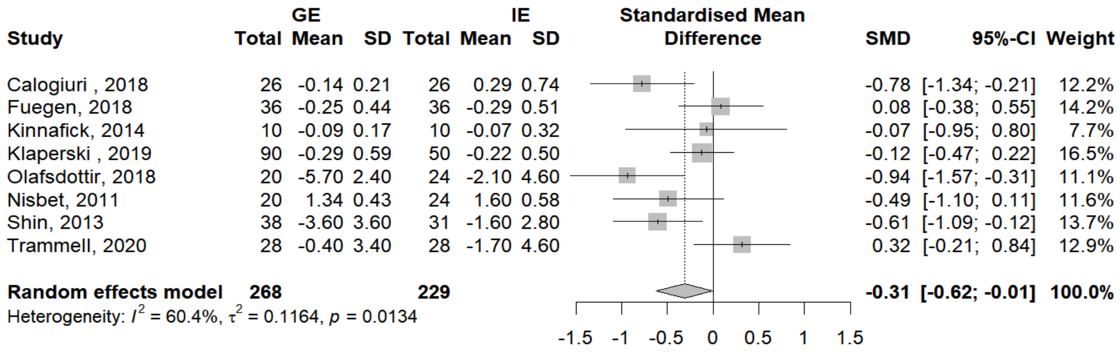

Fig S26. Forest plot of negitive affect: GE vs. IE

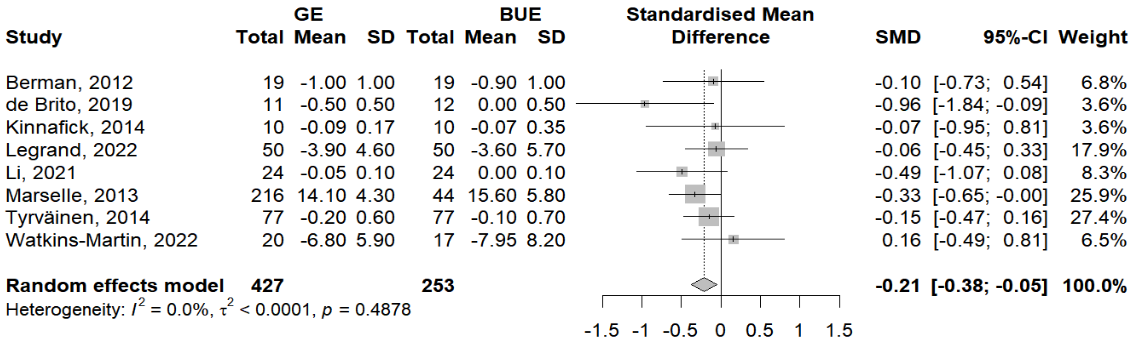

Fig S27. Forest plot of negitive affect: GE vs. BUE

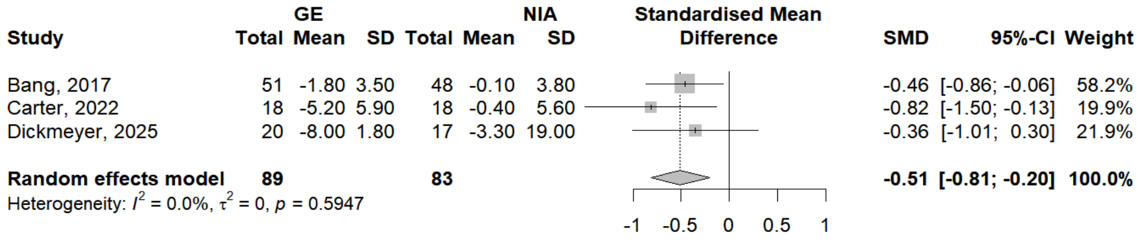

Fig S28. Forest plot of stress: GE vs. NIA

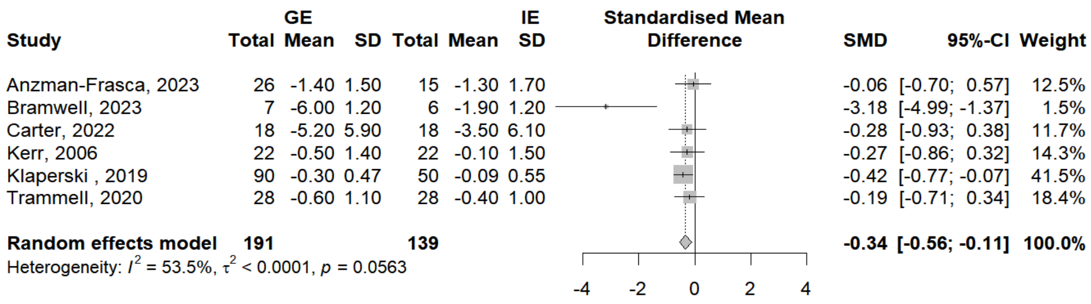

Fig S29. Forest plot of stress: GE vs. IE

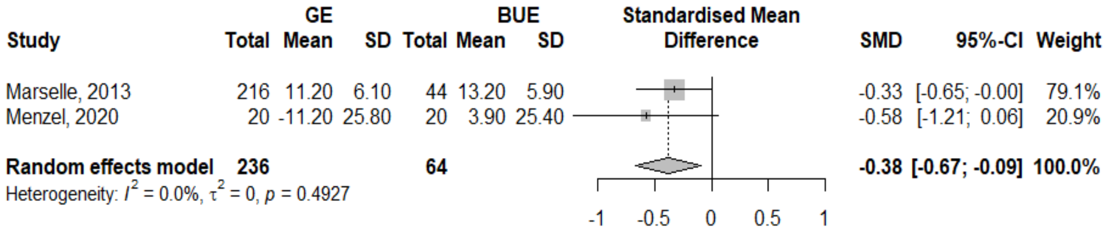

Fig S30. Forest plot of stress: GE vs. BUE

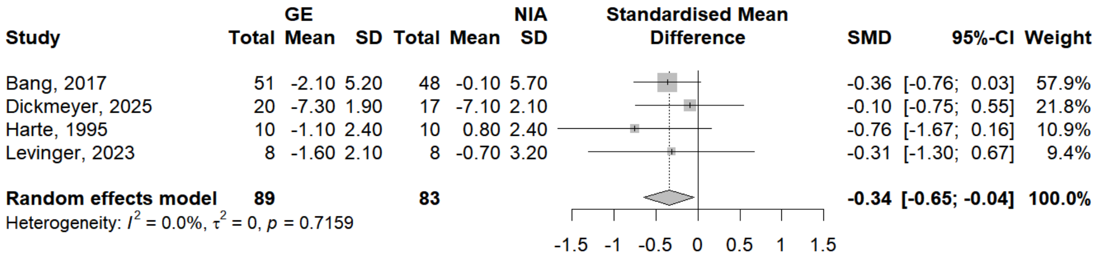

Fig S31. Forest plot of depression: GE vs. NIA

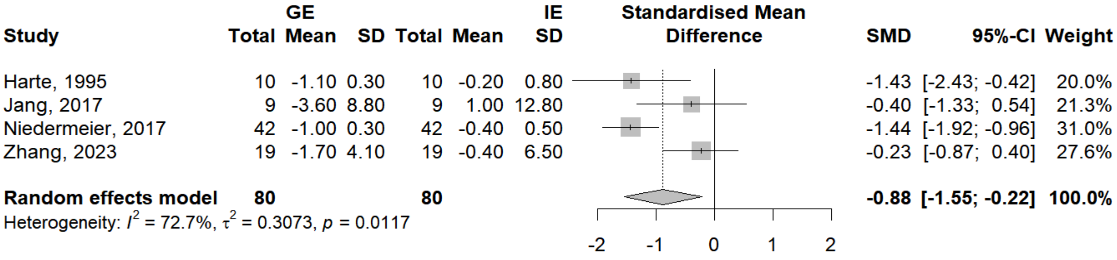

Fig S32. Forest plot of depression: GE vs. IE

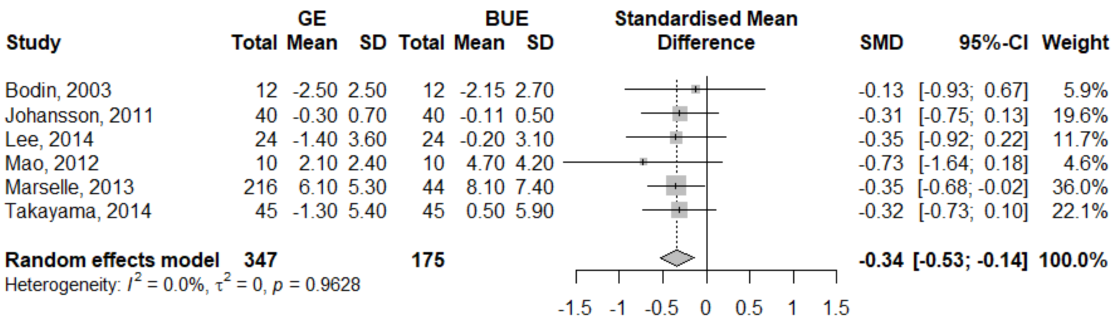

Fig S33. Forest plot of depression: GE vs. BUE

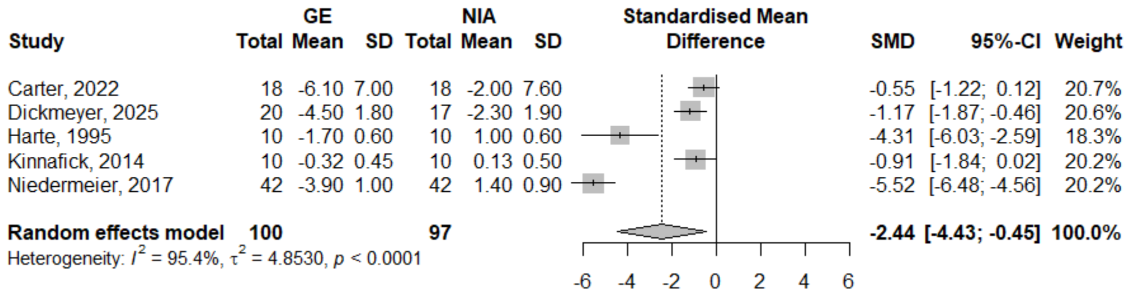

Fig S34. Forest plot of anxiety: GE vs. NIA

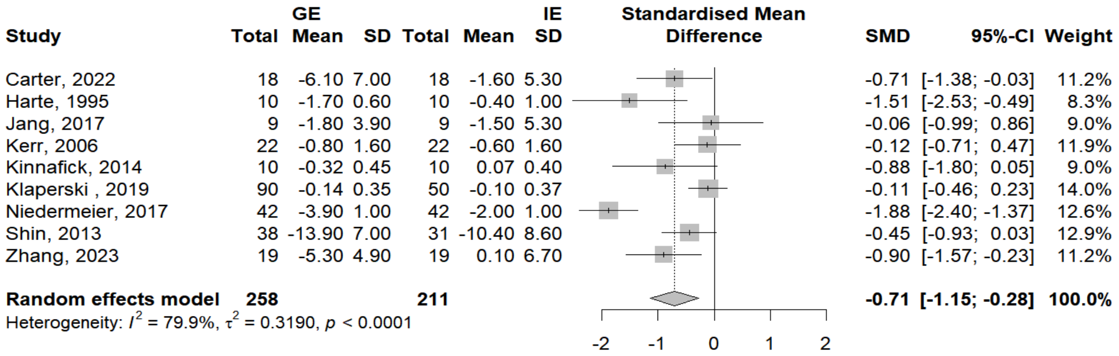

Fig S35. Forest plot of anxiety: GE vs. IE

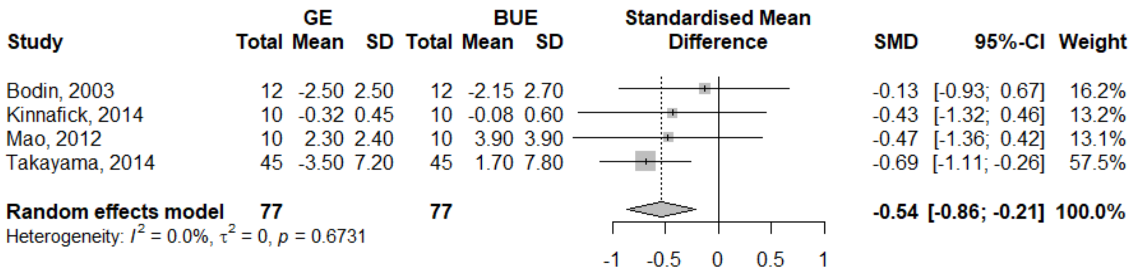

Fig S36. Forest plot of anxiety: GE vs. BUE

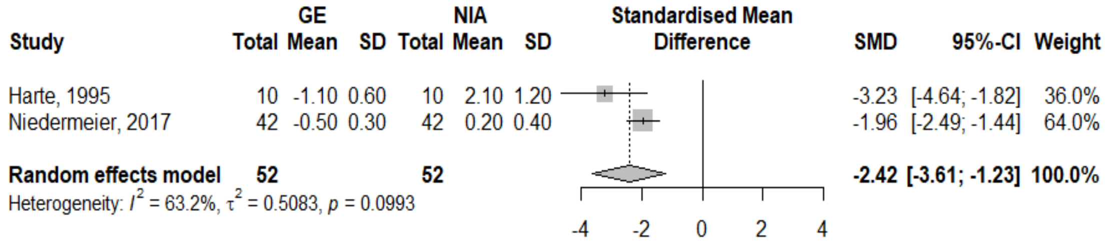

Fig S37. Forest plot of anger: GE vs. NIA

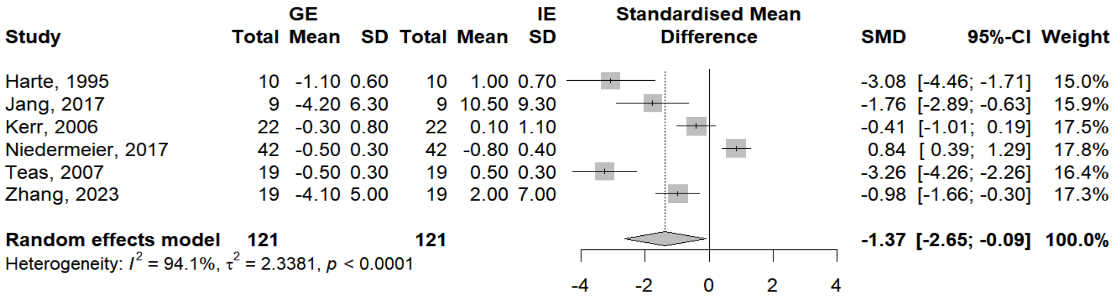

Fig S38. Forest plot of anger: GE vs. IE

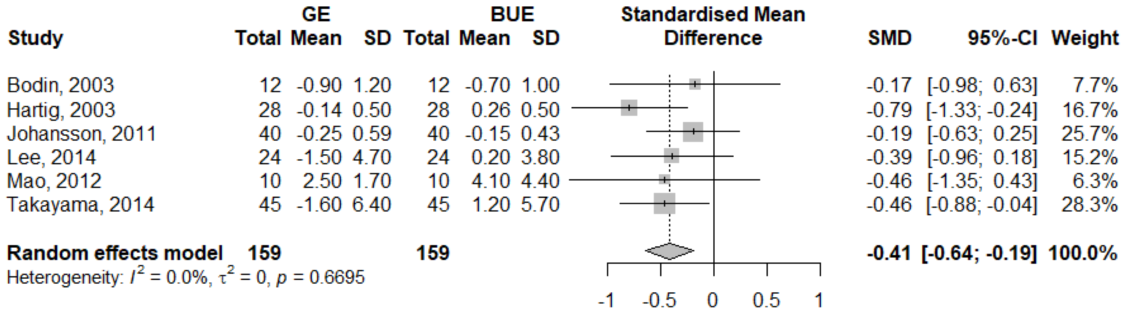

Fig S39. Forest plot of anger: GE vs. BUE

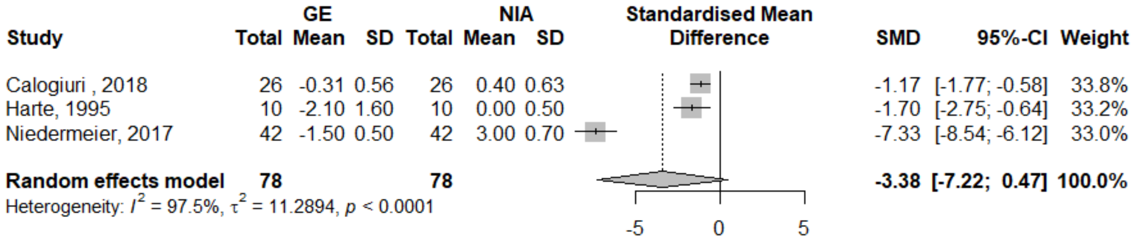

Fig S40. Forest plot of fatigue: GE vs. NIA

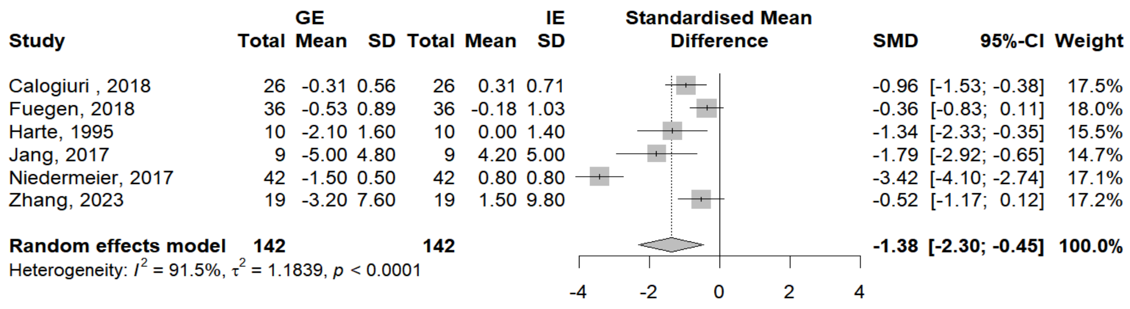

Fig S41. Forest plot of fatigue: GE vs. IE

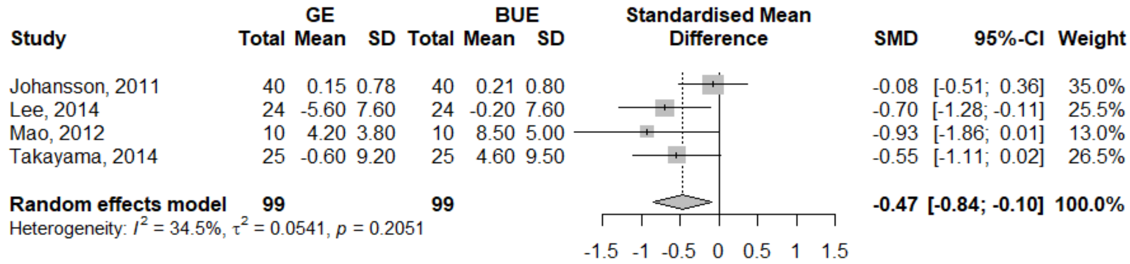

Fig S42. Forest plot of fatigue: GE vs. BUE

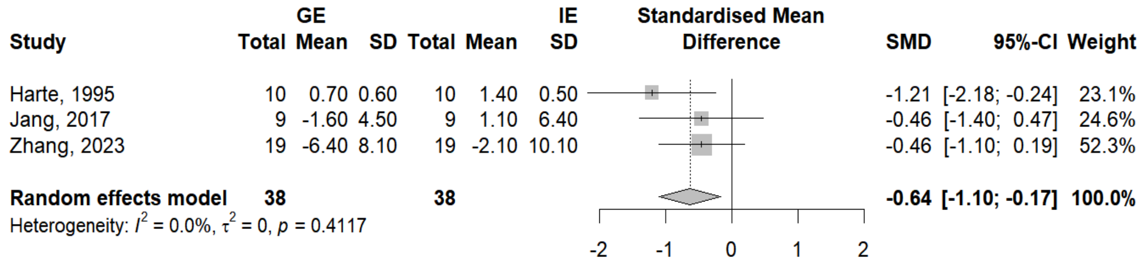

Fig S43. Forest plot of confusion: GE vs. IE

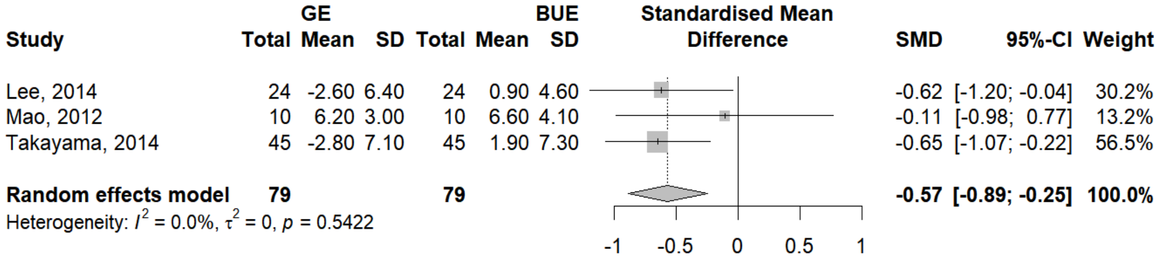

Fig S44. Forest plot of confusion: GE vs. BUE

S7 File Sensitivity Analysis

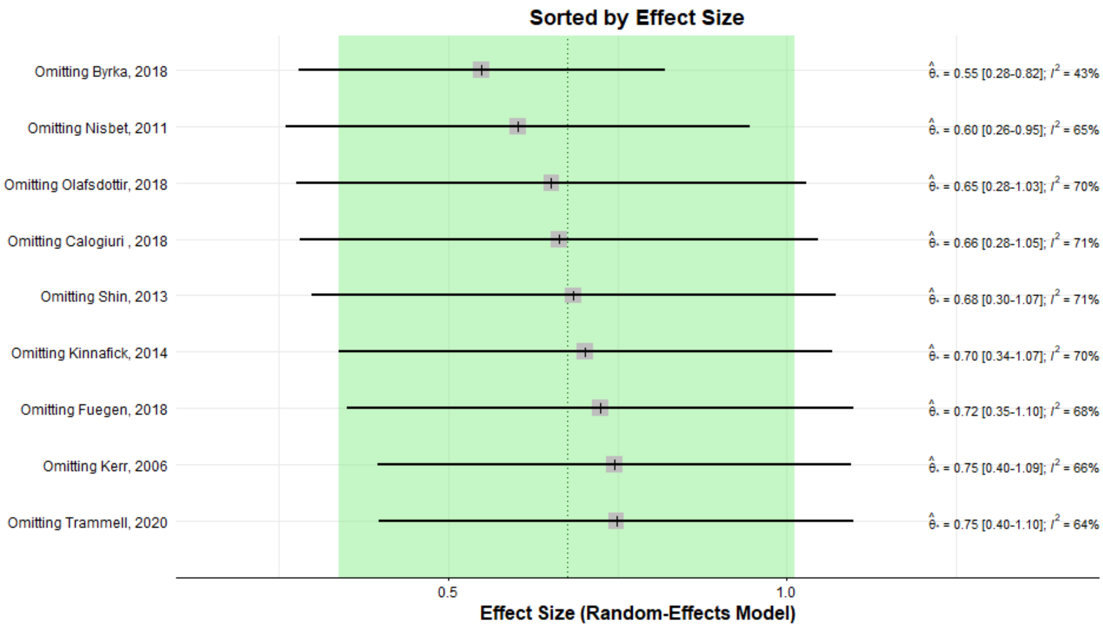

Fig S44. Sensitivity analysis of positive affect: GE vs. IE

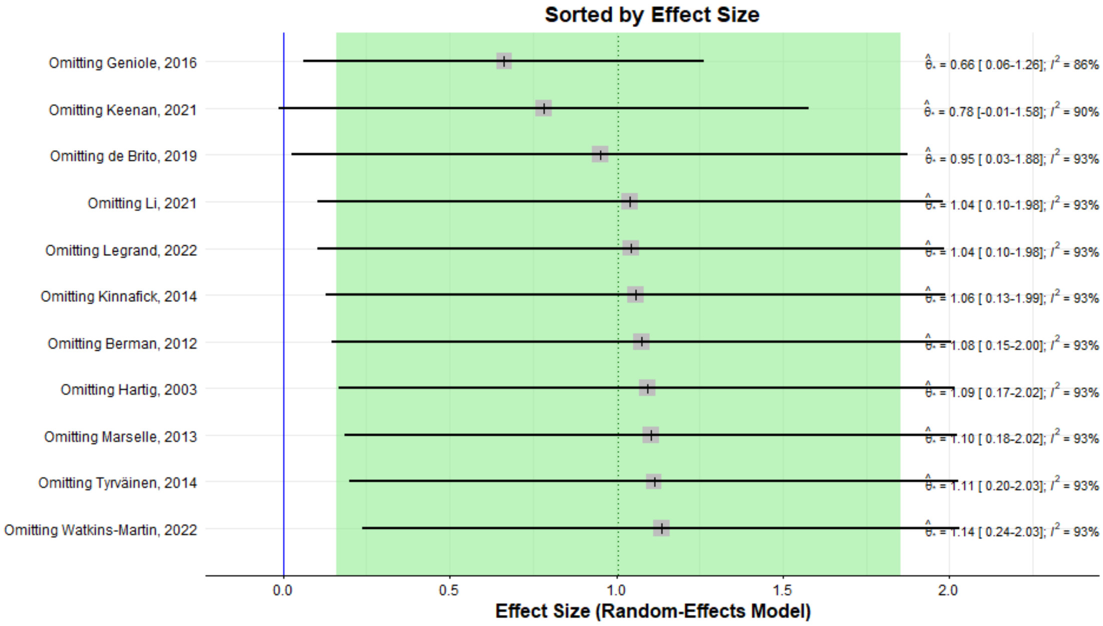

Fig S45. Sensitivity analysis of positive affect: GE vs. BUE

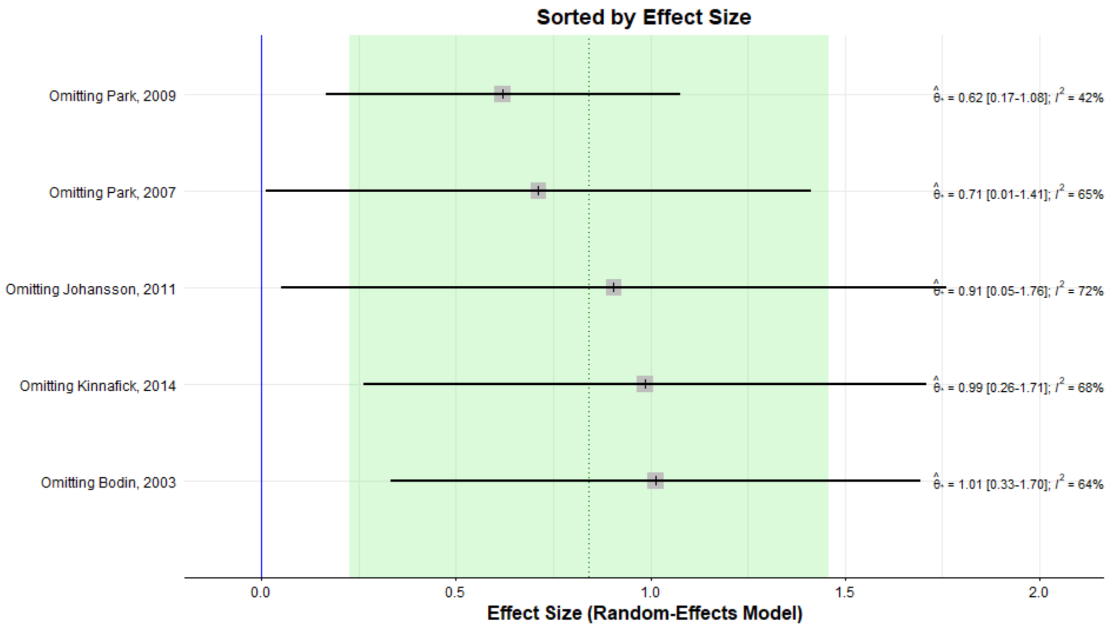

Fig S46. Sensitivity analysis of calm: GE vs. BUE

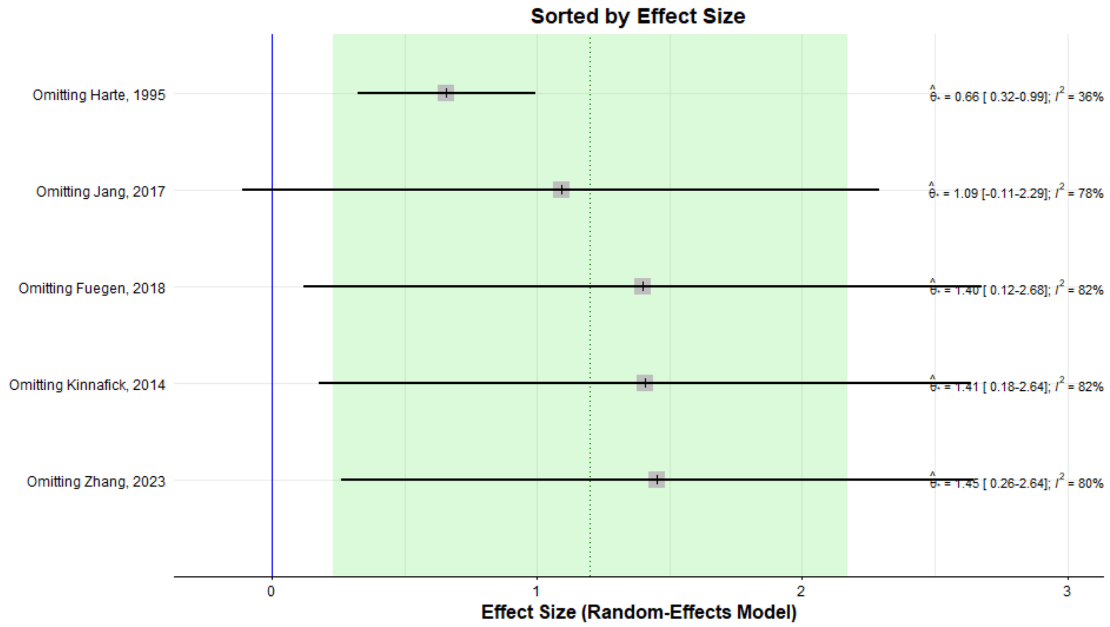

Fig S47. Sensitivity analysis of vigor: GE vs. IE

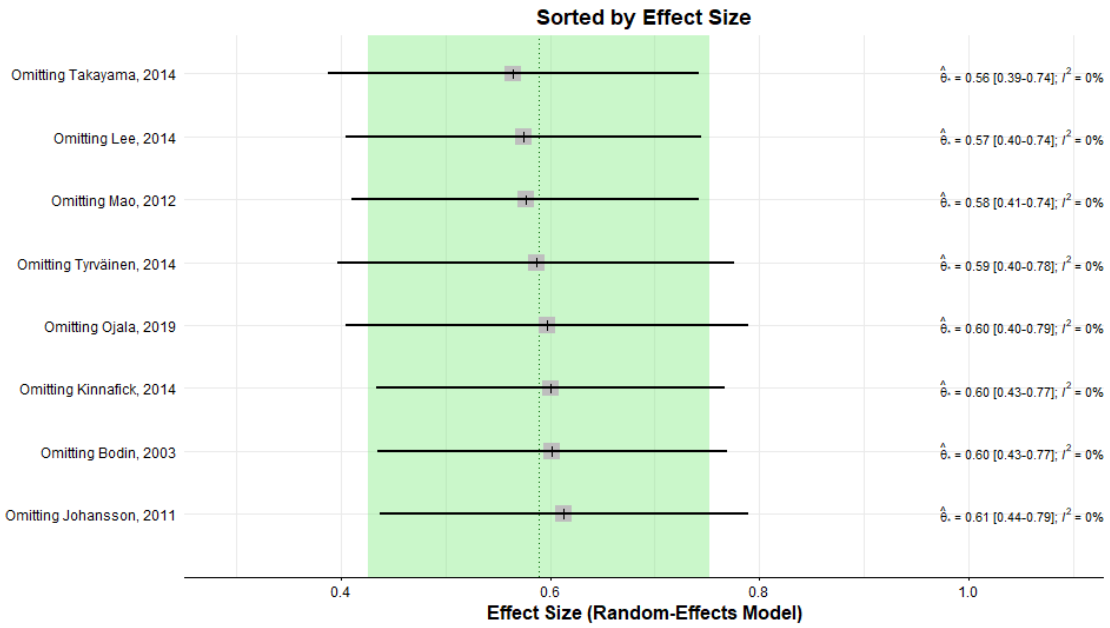

Fig S48. Sensitivity analysis of vigor: GE vs. BUE

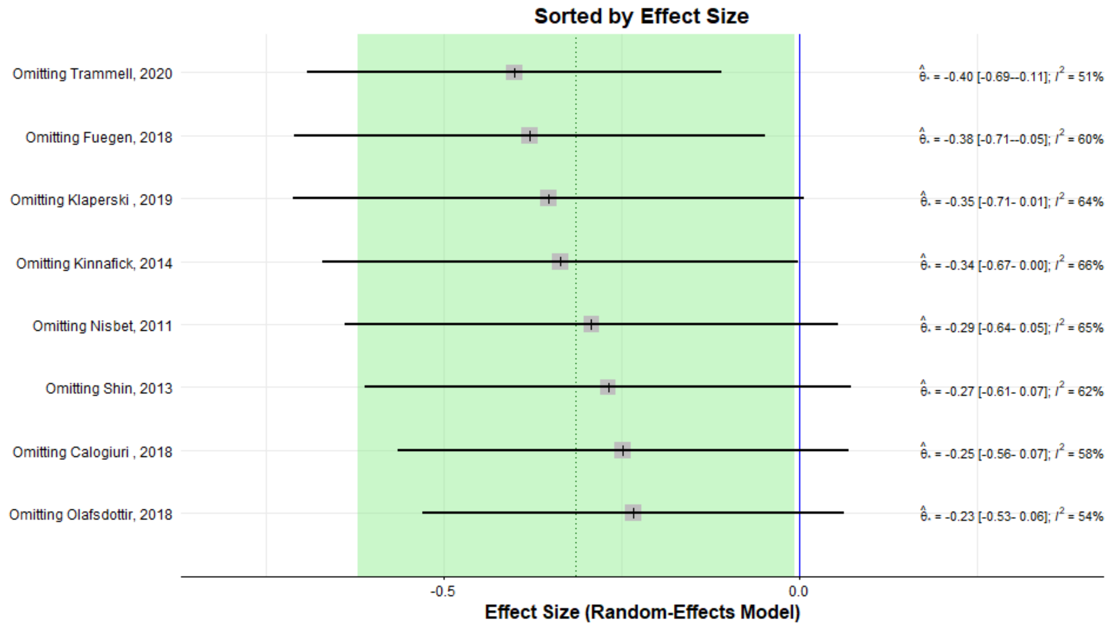

Fig S49. Sensitivity analysis of negative affect: GE vs. IE

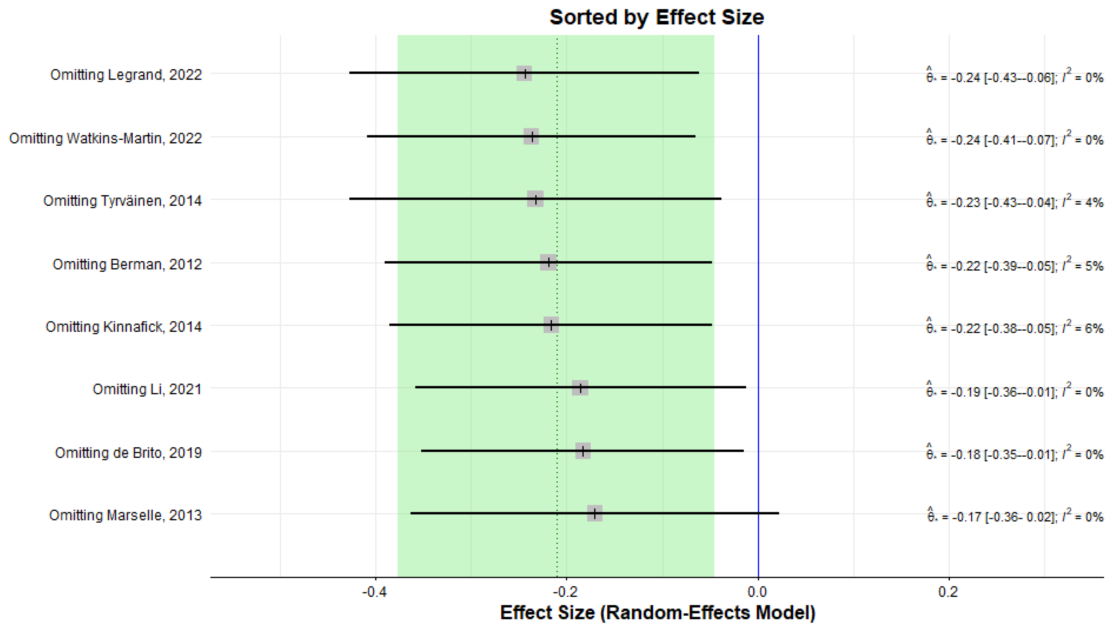

Fig S50. Sensitivity analysis of negative affect: GE vs. BUE

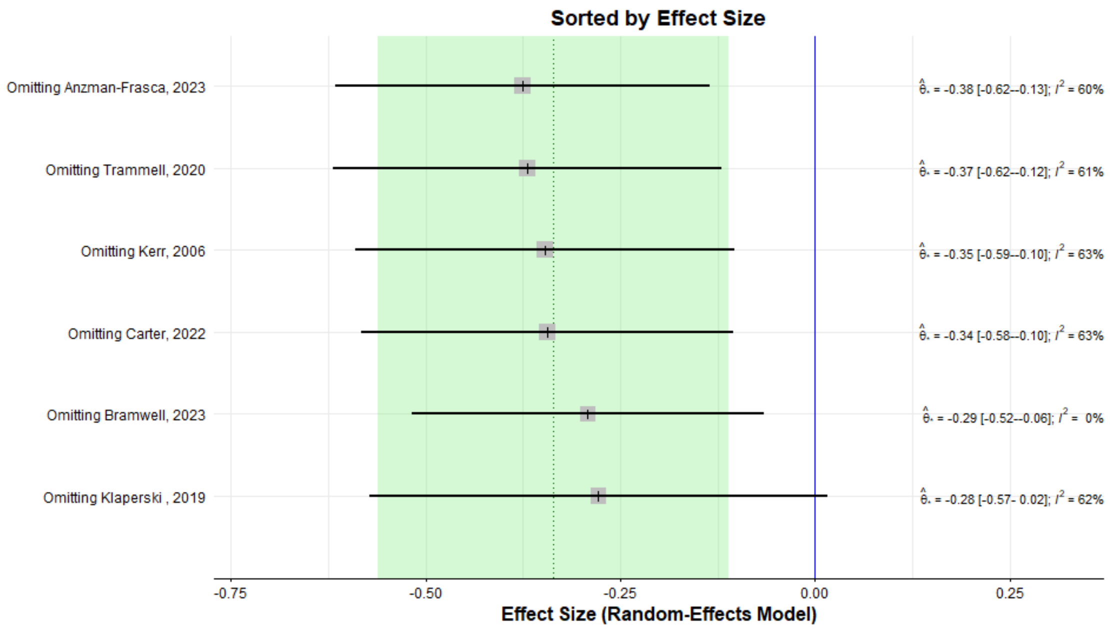

Fig S51. Sensitivity analysis of stress: GE vs. IE

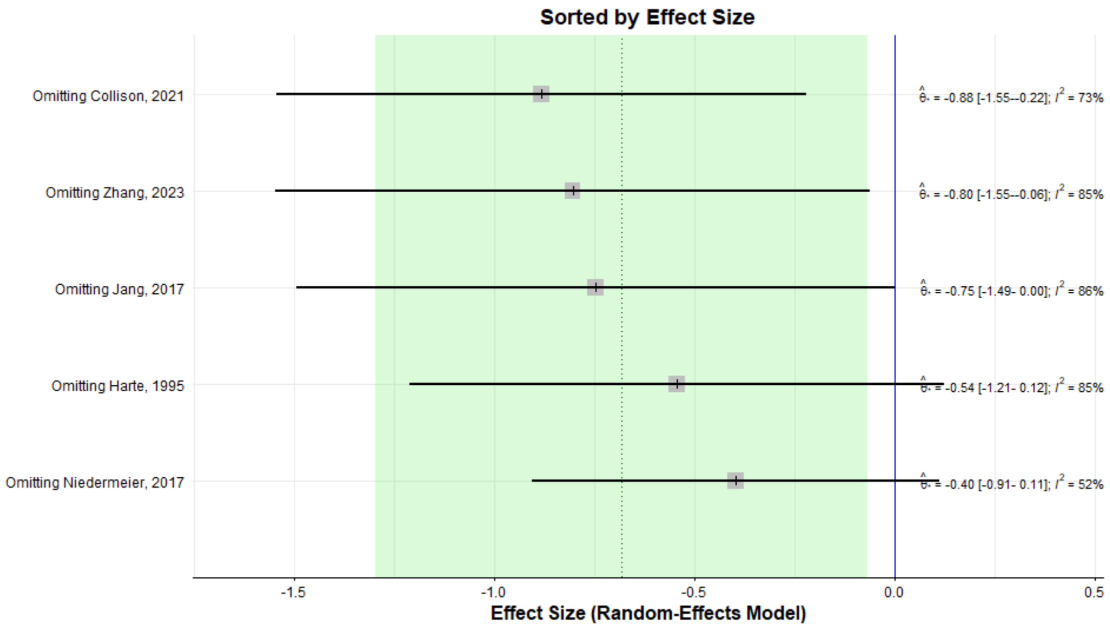

Fig S52. Sensitivity analysis of depression: GE vs. IE

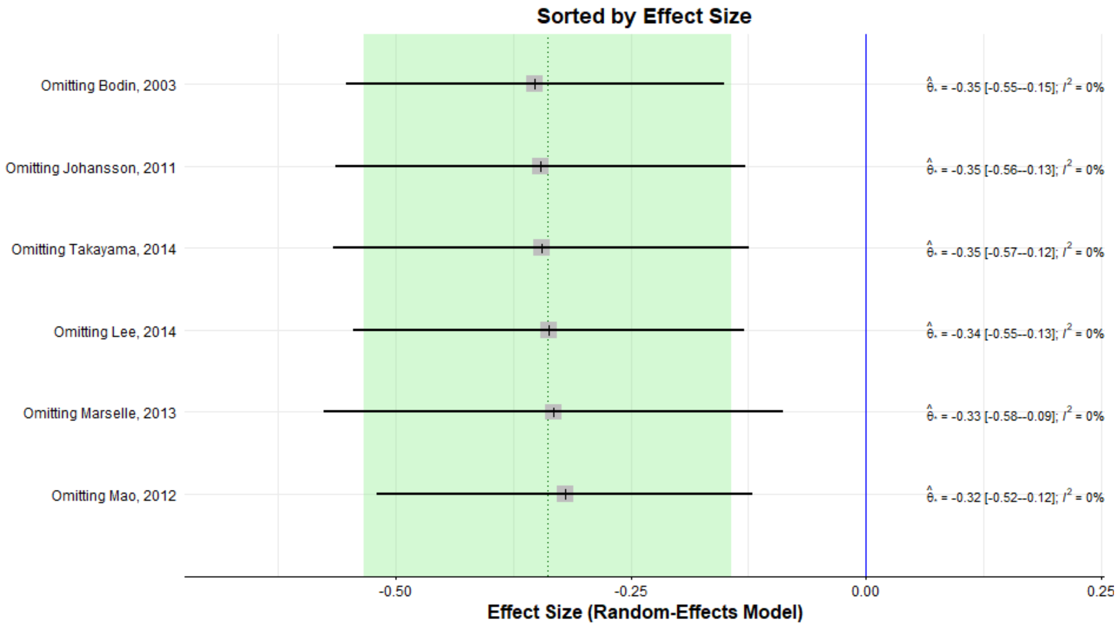

Fig S53. Sensitivity analysis of depression: GE vs. BUE

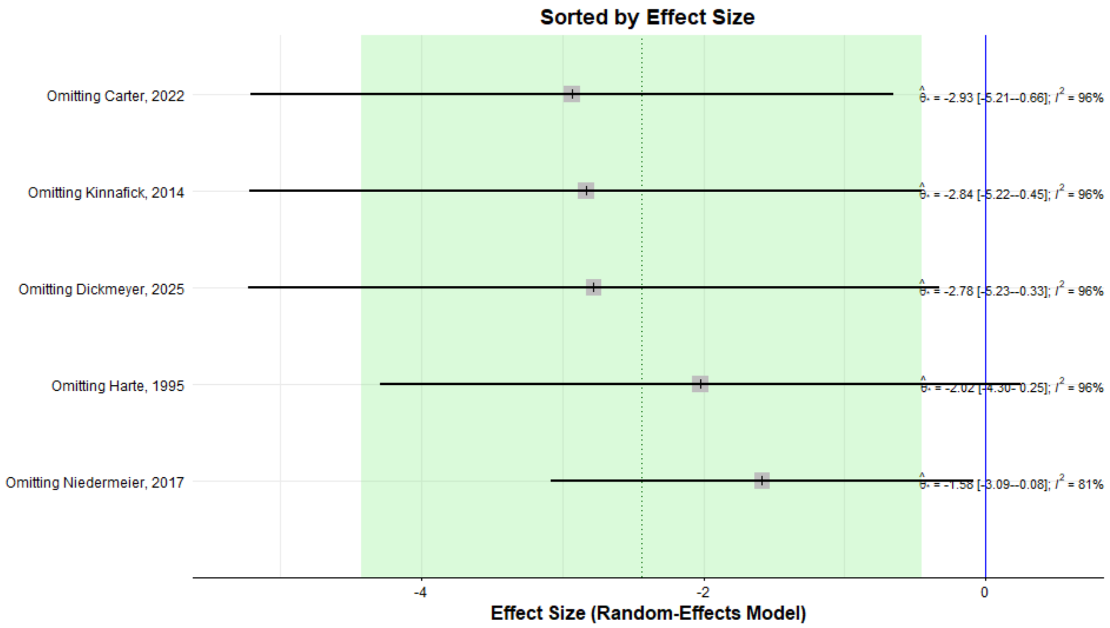

Fig S54. Sensitivity analysis of anxiety: GE vs. NIA

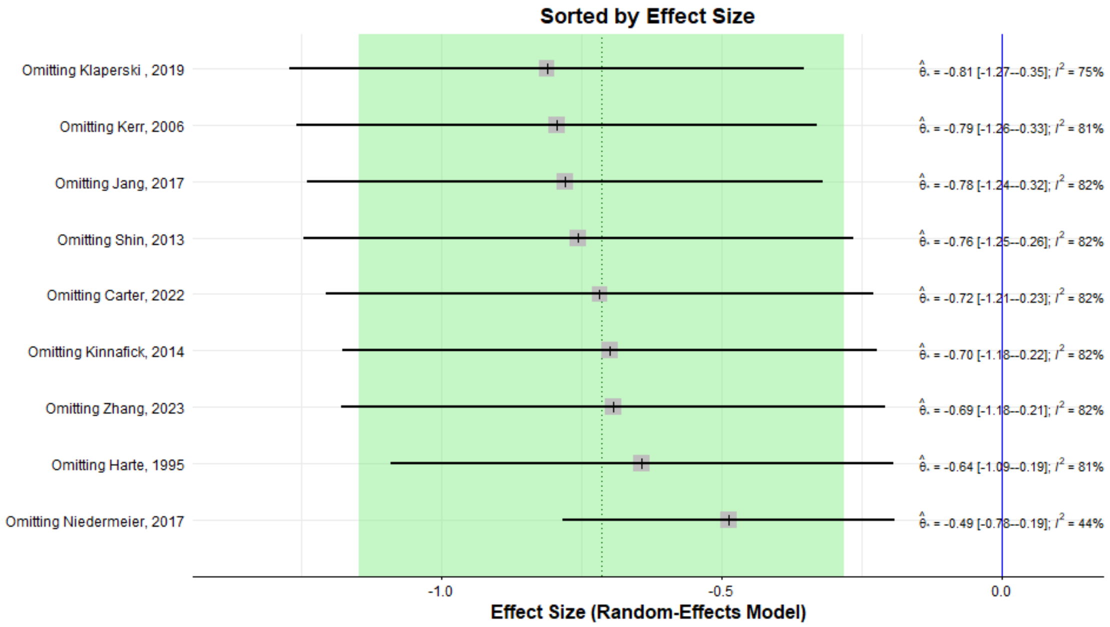

Fig S55. Sensitivity analysis of anxiety: GE vs. IE

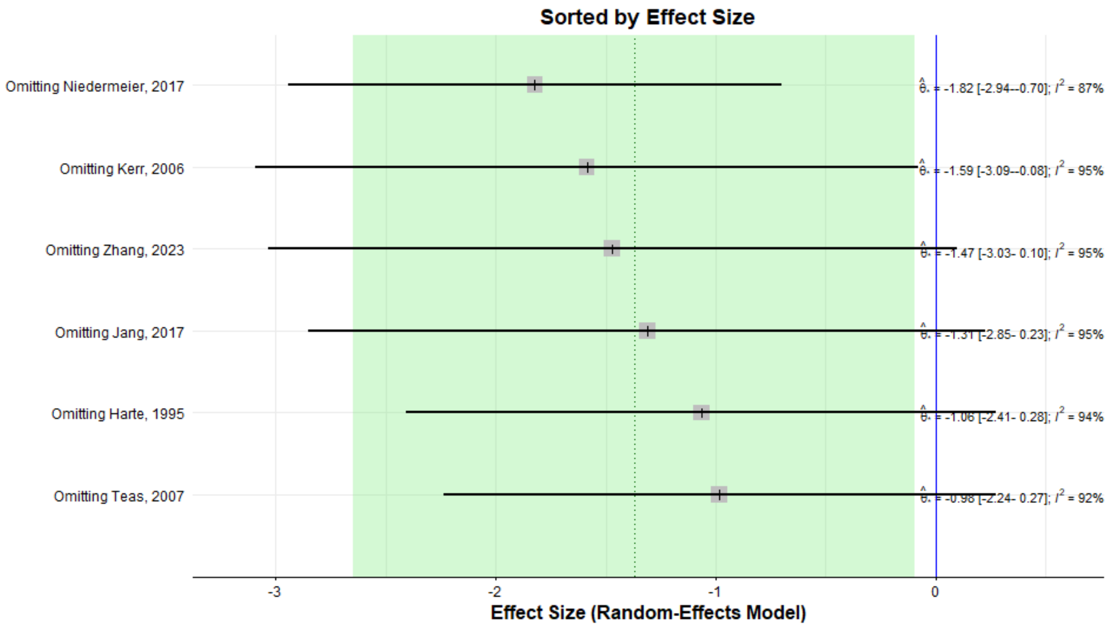

Fig S56. Sensitivity analysis of anger: GE vs. IE

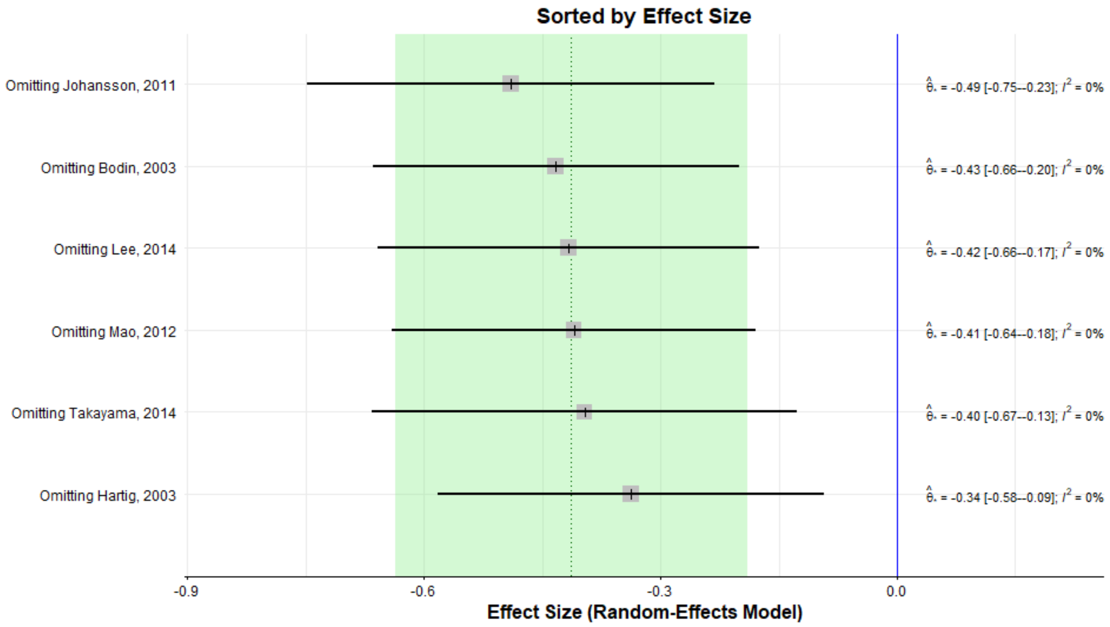

Fig S57. Sensitivity analysis of anger: GE vs. BUE

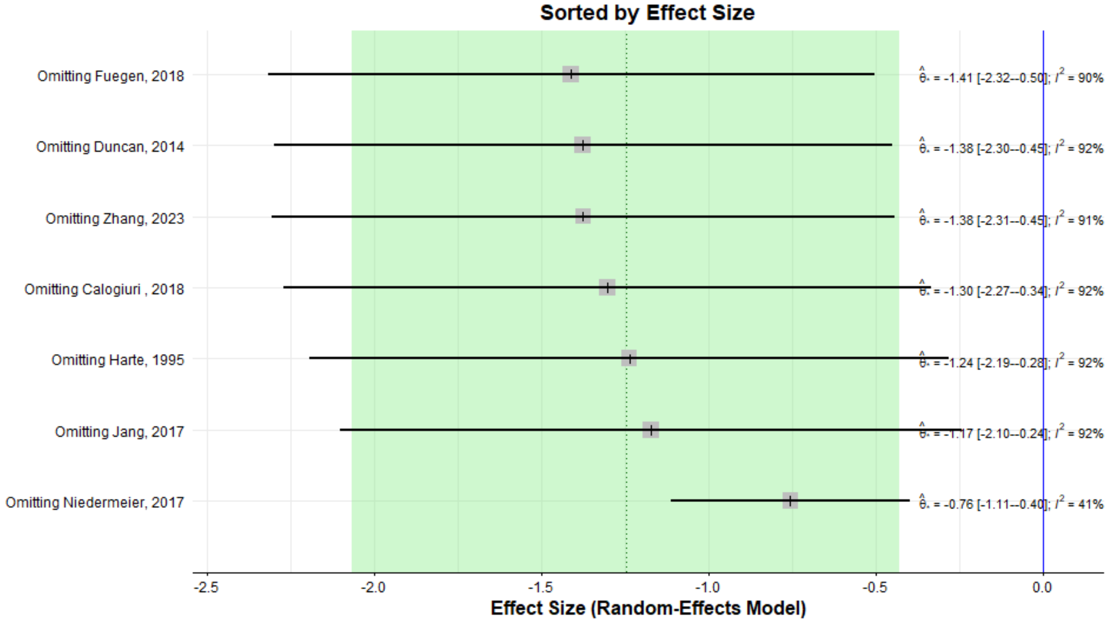

Fig S58. Sensitivity analysis of fatigue: GE vs. IE
